# Evaluating the persuasive influence of political microtargeting with large language models

**DOI:** 10.1073/pnas.2403116121

**Published:** 2024-06-07

**Authors:** Kobi Hackenburg, Helen Margetts

**Affiliations:** ^a^Oxford Internet Institute, University of Oxford, Oxford OX1 2JD, United Kingdom

**Keywords:** microtargeting, large language models, political persuasion, AI safety, AI-mediated communication

## Abstract

Recent advancements in large language models (LLMs) have raised the prospect of scalable, automated, and fine-grained political microtargeting on a scale previously unseen; however, the persuasive influence of microtargeting with LLMs remains unclear. Here, we build a custom web application capable of integrating self-reported demographic and political data into GPT-4 prompts in real-time, facilitating the live creation of unique messages tailored to persuade individual users on four political issues. We then deploy this application in a preregistered randomized control experiment (*n* = 8,587) to investigate the extent to which access to individual-level data increases the persuasive influence of GPT-4. Our approach yields two key findings. First, messages generated by GPT-4 were broadly persuasive, in some cases increasing support for an issue stance by up to 12 percentage points. Second, in aggregate, the persuasive impact of microtargeted messages was not statistically different from that of non-microtargeted messages (4.83 vs. 6.20 percentage points, respectively, *P* = 0.226). These trends hold even when manipulating the type and number of attributes used to tailor the message. These findings suggest—contrary to widespread speculation—that the influence of current LLMs may reside not in their ability to tailor messages to individuals but rather in the persuasiveness of their generic, nontargeted messages. We release our experimental dataset, *GPTarget2024*, as an empirical baseline for future research.

## Significance

Advances in large language models (LLMs) have raised concerns over scalable, personalized political persuasion. Here, we integrate user data into GPT-4 prompts in real-time, facilitating the live creation of messages tailored to persuade individual users on political issues. We then deploy this application at scale to test whether personalized, microtargeted messaging offers a persuasive advantage compared to nontargeted messaging. We find that while messages generated by GPT-4 were persuasive, in aggregate, the persuasive impact of microtargeted messages was not statistically different from that of nontargeted messages. These findings suggest—contrary to widespread speculation—that the influence of current LLMs may reside not in their ability to tailor messages to individuals but rather in the persuasiveness of their generic, nontargeted messages.

In 2023, the world witnessed an explosion in the accessibility and capabilities of AI in the form of large language models (LLMs). In 2024, over 4 billion people are expected to vote in what will be the largest election year in history. This confluence of events has caused great concern over the potential for AI technologies to disproportionately influence voters’ behavior and electoral outcomes (1, 2). However, even as researchers and industry leaders list model persuasiveness as a primary safety concern (3, 4), understandings of model capabilities in this domain remain nascent.

Early research suggests that most capable LLMs can directly persuade humans on political issues (5), draft more persuasive public communications than actual government agencies and political communication experts (6, 7), and generate convincing disinformation and fake news (1, 8, 9). Across these domains, humans are no longer able to consistently distinguish human and LLM-generated texts (8, 10). Crucially, these models are also highly scalable, allowing for essentially limitless production of political messages at an extremely low cost (2, 11).

In addition to quality and scalability, these models offer a third trait: the ability to personalize the style and content of their outputs via reinforcement learning from human feedback (RLHF), in-context learning, adaptive prompting, and flexible system instructions (12). This raises a clear worry: When integrated with existing databases of personal data, LLMs could tailor their messages to appeal to the vulnerabilities and values of specific individuals, potentially reinforcing their existing beliefs, or persuading them to adopt new ones (13–16). Experts have cautioned that these developments may open the door for a wide range of actors—even those without access to significant financial resources or technical expertise—to easily automate the generation of personalized political propaganda at a level of scale and quality previously unseen (11).

Concerns regarding personalized LLM-generated political content have renewed an already-existing fervor surrounding the practice of political microtargeting. In the years since the consulting firm Cambridge Analytica used Facebook data from 50 million voters to target political ads during the 2016 US presidential election, the media have written extensively about the capacity of personalized, data-driven influence operations to sway citizens’ political opinions, influence elections, and damage democratic institutions (17–22).

Despite the hype and scrutiny, the efficacy of political microtargeting techniques has been difficult to establish. Political microtargeting relies on treatment effect heterogeneity—different groups of people responding in different ways to different messages. However, a body of research suggests that such divergent reactions are rare, and that persuasive political messages tend to sway people, regardless of their demographic traits, in broadly similar ways (23–25). Several studies on microtargeting found inconclusive or underwhelming evidence supporting its effects (26, 27).

Recent empirical research paints a more nuanced picture, suggesting that political microtargeting may indeed enhance the persuasiveness of political campaigns. The most extensive evaluation to date, conducted by Tappin et al., suggests that microtargeting based on demographic and political attributes does present a significant advantage over other nontargeted messaging strategies, although this advantage is issue-specific (28). This finding builds on prior work suggesting that political advertisements have increased persuasive power when tailored to an individual's position on the introversion–extroversion spectrum (29) or other psychological characteristics (30). These studies reflect a growing empirical consensus that political microtargeting can under some circumstances be an effective means of exerting persuasive influence.

However, in spite of the apparent efficacy of political microtargeting in some contexts and the flexible, scalable nature of LLM-generated content, the persuasive influence of political microtargeting with LLMs could be limited. The ability of a LLM to leverage demographic attributes to persuasive effect depends on its ability to accurately encode and reflect the beliefs, opinions, and values of that group. However, it remains unclear whether the best currently available LLMs can accurately map a demographic attribute (or set of attributes) to corresponding political opinions or preferences (31).

LLMs such as GPT-3 and GPT-3.5 have shown some capability to mirror the attitudes of certain political groups. For instance, when prompted to adopt a liberal or conservative political identity, these models produce text reflecting the respective moral biases (32). Further, GPT-2, when fine-tuned on a dataset of tweets from liberal and conservative Twitter users, was able to reflect partisan views more accurately, surpassing several baseline methods in terms of alignment (33). A recent study also found evidence that GPT-3 is capable of replicating viewpoints—such as presidential candidate preference—of some demographically varied subpopulations within the United States (34).

However, recent research has also found substantial misalignment between the opinions reflected by current LLMs and those of fine-grained demographic groups in the United States, even when the model is prompted to role-play as a member of the group. An evaluation of nine major publicly accessible LLMs (including six from OpenAI) across 22 US demographic groups found that no models were able to accurately represent the actual political opinion distributions of these groups. Furthermore, these models struggled to represent nuanced political views consistently and were minimally adjustable via prompting (31).

This means that even as scholars, policymakers, and technologists have jointly underscored the potential for the massive scaling of automated, persuasive political microtargeting (2), the capabilities of LLMs in this domain are uncertain, untested, and poorly understood. In the present work, we aim to fill this gap, quantifying the extent to which microtargeting with the most powerful publicly accessible LLM—GPT-4—can enhance its persuasive influence on political issues. We operationalize this broader research aim via three preregistered research subquestions:

RQ1: Are messages generated by an LLM with access to data about the demographic and political attributes of their audience more persuasive than messages generated by an LLM without access to this data?

RQ2: Are messages generated by an LLM with access to more data about the demographic and political attributes of their audience more persuasive than messages generated by an LLM with access to less data?

RQ3: Do different political and demographic attributes, when used to tailor a message, have varying degrees of impact on the persuasive influence of that message?

To answer these questions, we develop a custom web application allowing for the injection of self-reported demographic and political data into GPT-4 prompts in real time. We then use this application to conduct a large, randomized human-subjects experiment, facilitating the live creation of thousands of unique messages tailored to persuade individual participants on political issues.

This work makes theoretical, methodological, and empirical contributions to the study of LLM safety, AI-mediated communication, and political influence. Theoretically, this study addresses the ambiguity surrounding the relationship between political persuasion, microtargeted messaging, and AI capabilities, providing a more precise estimate of the persuasive impact of AI-driven political microtargeting than has been available to date. Methodologically, it offers a robust and replicable approach—through a custom web-based pipeline—to integrating LLMs into experimental designs, paving the way for future research evaluating personalized LLMs. Empirically, it contributes a novel dataset, *GPTarget2024*, containing metadata for thousands of microtargeted messages generated by GPT-4. Taken together, these contributions evaluate a critical aspect of AI's potential impact on the political public sphere and offer evidence to policymakers, technologists, and the wider public.

## Results

**Fig. 1.**
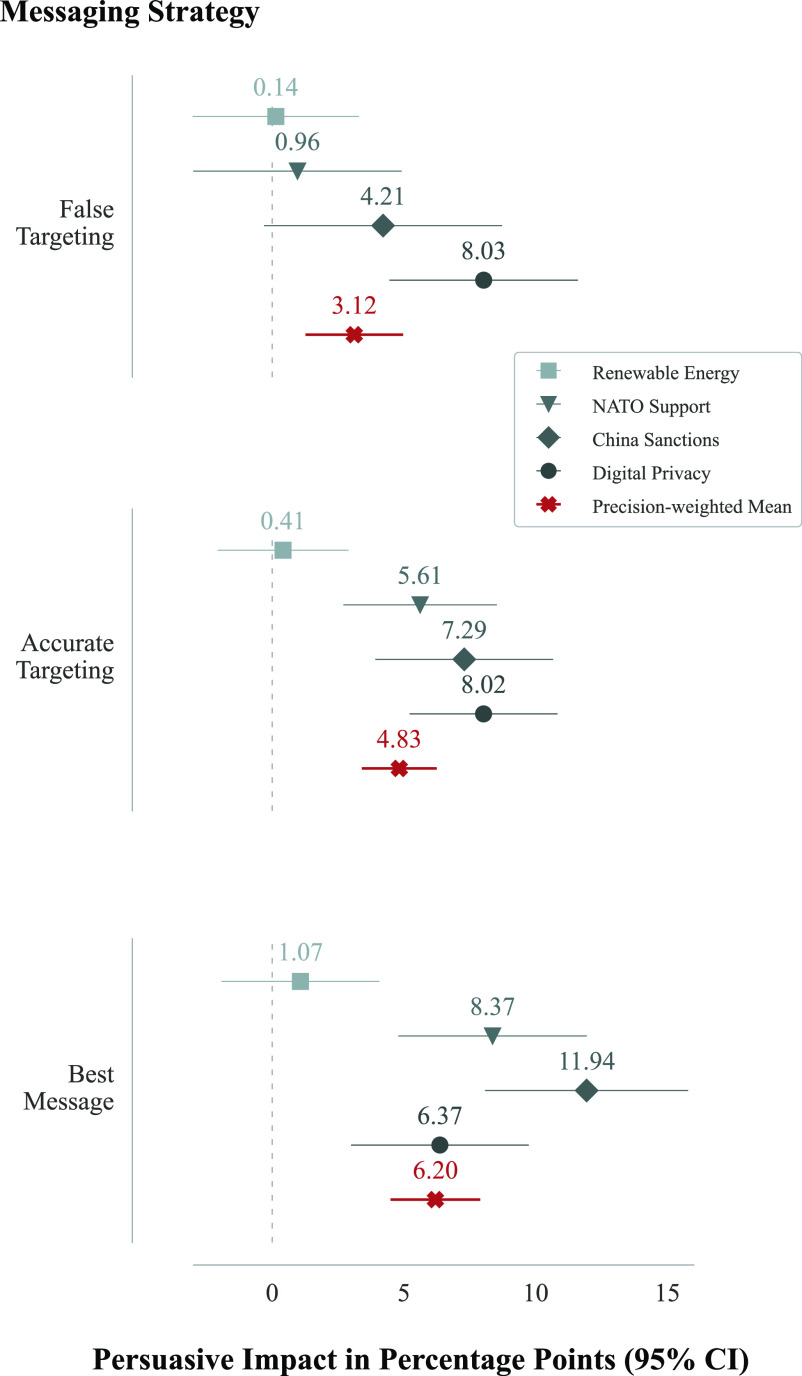
Political microtargeting does not enhance the persuasive influence of GPT-4 relative to nontargeted messages. The first row displays the estimated persuasive impact of a message tailored to incorrect attributes (the false targeting condition), the second displays the estimated persuasive impact of messages tailored to correct attributes (the accurate targeting condition) and the third displays the estimated persuasive impact of a nontargeted message (the best message condition). For the two targeting conditions, the estimated persuasive impact is collapsed across all levels of microtargeting and targetable attributes. Average issue stance alignment across conditions can be found in *SI Appendix*, Table S1.

*RQ1* concerned the extent to which messages generated by an LLM *with* access to political and demographic attributes about their audience were more persuasive than messages generated by an LLM *without* access to this data. As shown in Fig. 1, on average (across all levels of microtargeting and across all targetable attributes), while each treatment condition produced messages which were persuasive with respect to a control group, the results do not demonstrate a persuasive advantage of accurate microtargeting over the best message (non-microtargeting) condition. All reported estimates and *P*-values are based on OLS regression models with robust SE.

On digital privacy, the persuasive impact of accurate targeting was not statistically different from the best message condition (8.02 vs. 6.37 percentage points, respectively, *P* = 0.171) or the false targeting condition (8.02 vs. 8.03, respectively, *P* = 0.997). On China sanctions, the estimated persuasive impact of accurate targeting was about 40% weaker than the best message condition (7.29 vs. 11.94, respectively, *P* < 0.001) and not statistically different from the false targeting condition (7.29 vs. 4.21, respectively, *P* = 0.082). On NATO support, the estimated persuasive impact of accurate targeting was about 33% weaker than the best message condition (5.62 vs. 8.37, respectively, *P* < 0.042) but significantly stronger than the false targeting condition (5.62 vs. 0.96, respectively, *P* < 0.001). On renewable energy, the persuasive impact of accurate targeting was not statistically different from the best message condition (0.41 vs. 1.07, respectively, *P* = 0.547) or the false targeting condition (0.41 vs. 0.14, respectively, *P* = 0.818). Notably, the estimated effects of all treatment conditions on the renewable energy issue were negligible and statistically insignificant when compared to the control group. This issue also had extremely high initial issue support, which may have contributed to the lack of significant treatment effects; see *SI Appendix*, Fig. S4 and Table S2.

To assess the overall impact of microtargeting (as opposed to the effect of microtargeting on a particular policy issue), the precision-weighted mean across all issues was computed. In aggregate, the results found that the average persuasive impact of the accurate targeting condition did not differ statistically from that of the best message condition (4.83 vs. 6.20 percentage points, respectively, *P* = 0.226) or the false targeting condition (4.83 vs. 3.12, respectively, *P* = 0.152). The persuasive impact of the best message condition was approximately twice as large the false targeting condition (6.20 vs. 3.12, respectively, *P* = 0.016). We report these results disaggregated by demographic traits in *SI Appendix*, Figs. S5–S10, finding no significant differences in the effect of microtargeting with respect to gender, education, political affiliation, level of political engagement, or religious affiliation. However, we do find significant differences with respect to age: people aged 46 or older were significantly more persuaded by the microtargeted messaging than those in younger age brackets.

**Fig. 2.**
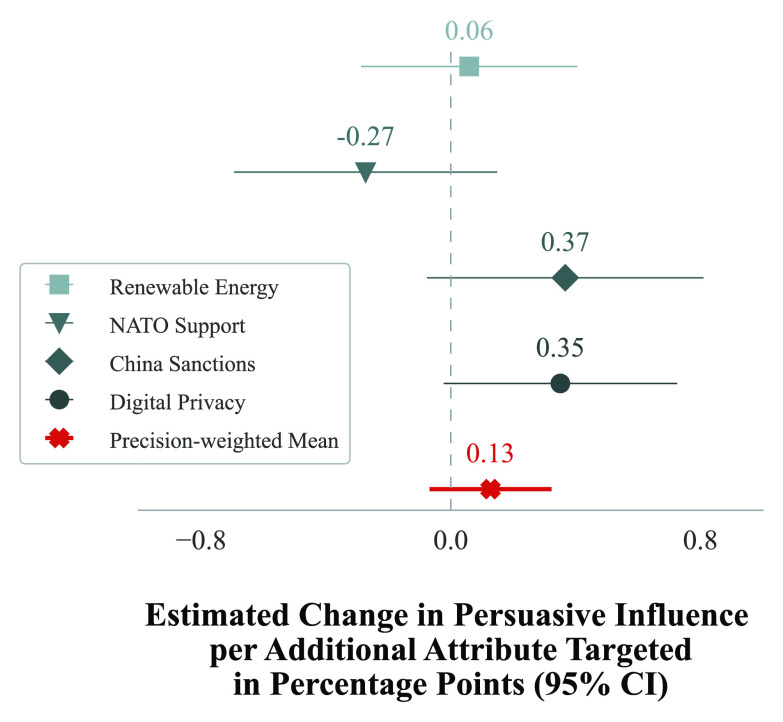
On average, increasing the number of attributes used by GPT-4 to tailor a message does not alter its persuasive impact. The first four rows display the estimated change in persuasive influence per additional attribute added for a given issue; the final row displays the mean across all issues.

*RQ2* concerned the extent to which messages generated by an LLM with access to *more* demographic and political attributes caused greater attitude change than messages generated by an LLM with access to *fewer* attributes. In aggregate, the results shown in Fig. 2 offer limited evidence that tailoring based on more attributes is associated with greater persuasive influence: the expected change in persuasive impact for each additional attribute used to tailor the message was not statistically different from 0 at the issue level (0.06 percentage points for renewable energy, *P* = 0.738; −0.27 for NATO support, *P* = 0.205; 0.37 for China sanctions, *P* = 0.105; 0.35 for digital privacy, *P* = 0.066) or when averaged across issues (0.13 percentage points, *P* = 0.20).

**Fig. 3.**
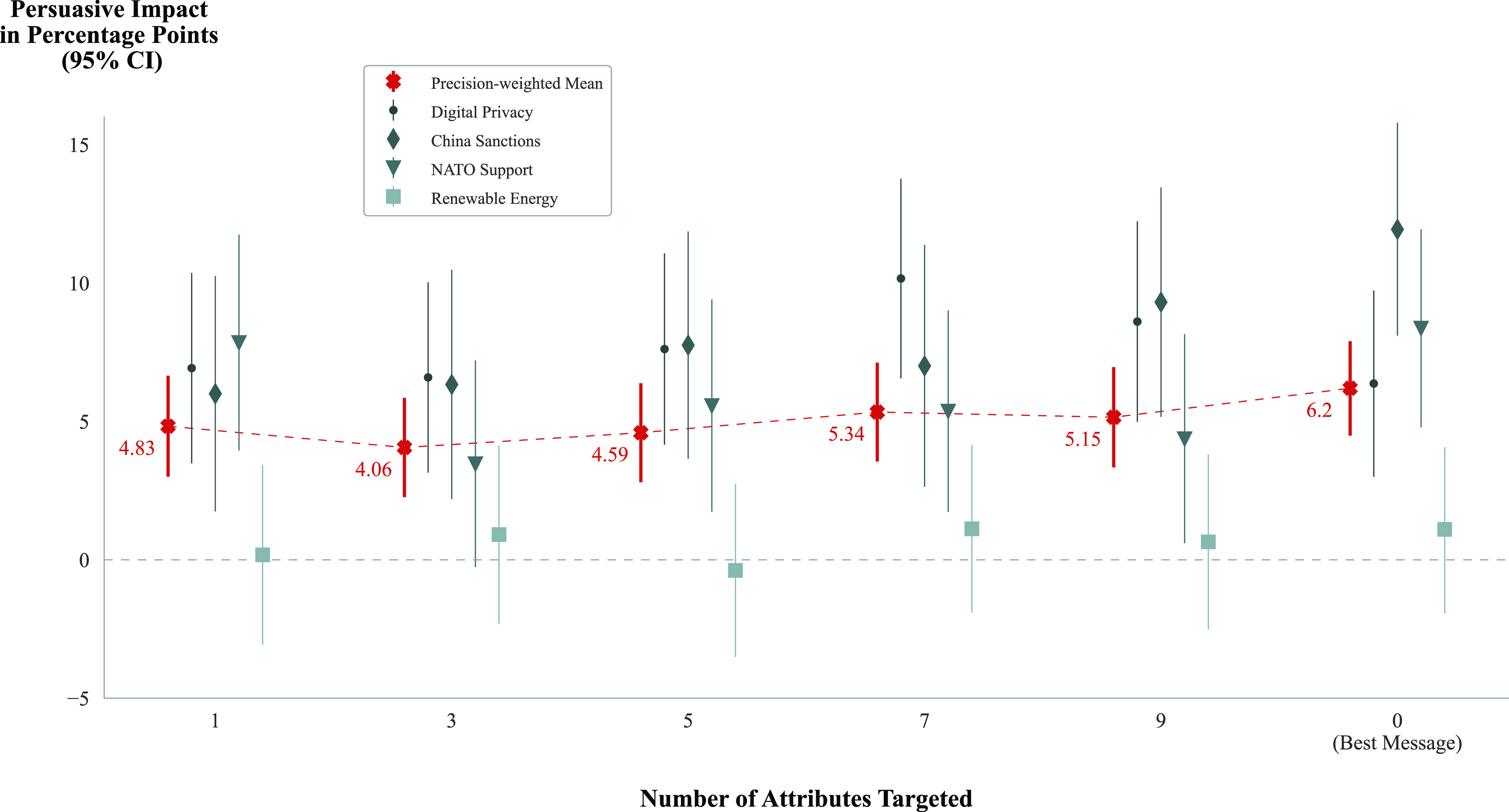
Mean persuasive influence for messages at each accurate targeting subcondition. Averaged across all issues, the expected change in persuasive impact for a message tailored based on a single attribute was not significantly less than that of a message tailored on nine attributes. The results for the best message condition are plotted on the far right.

In order to investigate the possibility that the relationship between the number of attributes used to tailor the message and the persuasive influence of that message was nonlinear and to provide a more detailed look at the effects of each level of targeting, a second model was fit. This model examined the differences in means between the five subconditions in the accurate targeting condition. These subconditions correspond to the number of attributes used by the model to tailor the generated message. The results, shown in Fig. 3, largely confirm the negligible effects illustrated by the first model. They show that in aggregate across all issues, the expected change in persuasive impact for a message targeted based on a single attribute was not significantly less than that of a message targeted on nine attributes (*P* = 0.805).

**Fig. 4.**
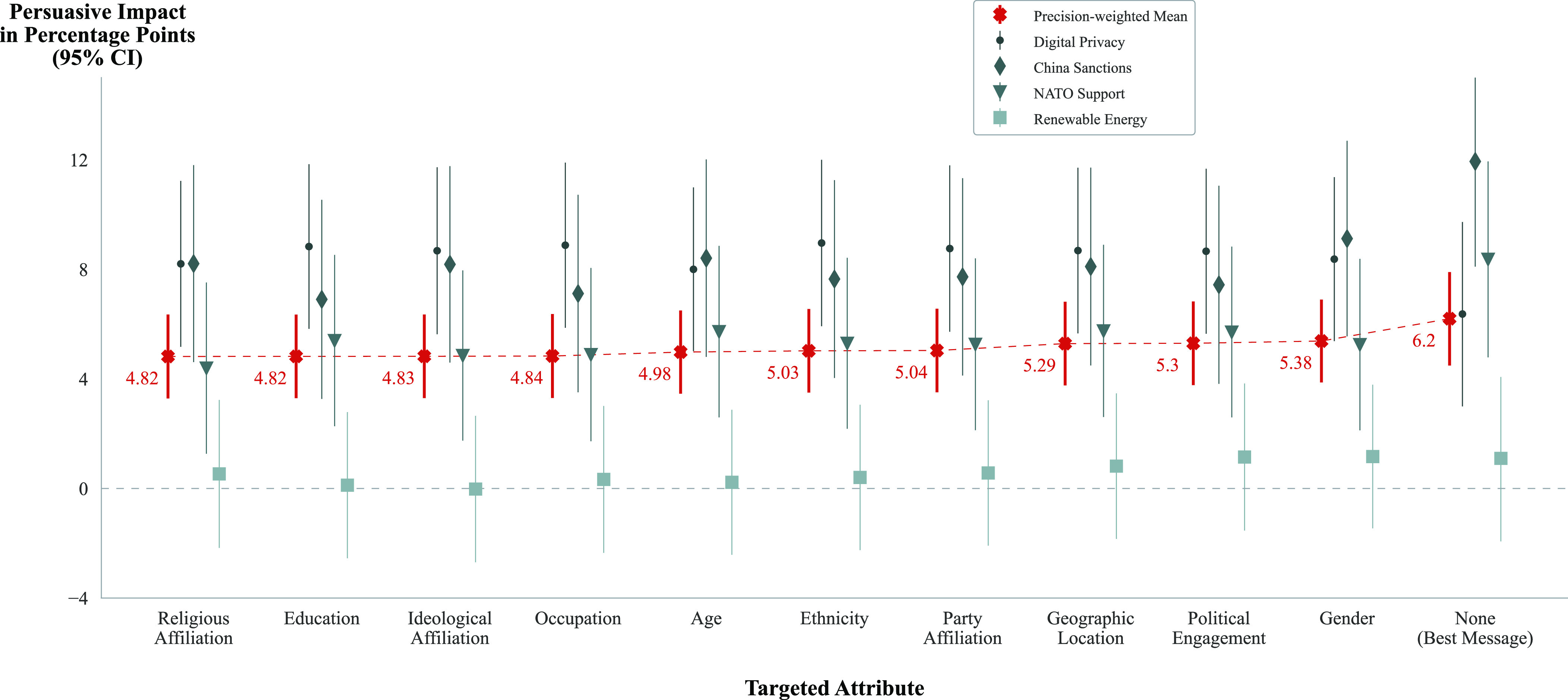
Mean persuasive influence for messages tailored using a given attribute. Within and across issues, no attribute was significantly more or less persuasive than any other when used to tailor a message.

*RQ3* asked the extent to which different political and demographic attributes, when used to tailor a message, had varying degrees of impact on the persuasive power of that message. In aggregate, the results shown in Fig. 4 offer limited evidence that targeting using particular attributes produced more persuasive messages. Targeting with a given attribute did not offer a persuasive advantage compared to targeting using any other attribute for renewable energy (*P* = 0.714), NATO support (*P* = 0.793), China sanctions (*P* = 0.183), digital privacy (*P* = 0.885), or in aggregate (*P* = 0.689).

We also report here the results from several posttreatment questions, which participants completed after providing a dependent variable response. First, participants were asked to report who they thought was most likely the author of the message they were exposed to. Participants in the accurate targeting condition were 3 percentage points more likely to identify the message as AI-generated compared to the best message condition (*P* = 0.012). For a full list of responses to the authorship question, see *SI Appendix*, Table S3.

As an additional measure of construct validity, participants were also asked who they thought would find the message they were shown most compelling, in terms of similarity to themselves. This question aimed to assess whether participants who received the messages tailored to their attributes actually perceived the message as likely to be persuasive to someone like themselves. Participants thus rated the message they were shown on a scale from “persuasive to someone very different from me” to “persuasive to someone very similar to me,” where similarity was explicitly defined as sharing political and demographic attributes.

The results suggest that to a marginal extent, tailored messages were indeed perceived by participants as being persuasive to self-similar others: Participants in the accurate targeting condition who were shown messages tailored to some combination of their attributes were statistically more likely to say that the messages would be most compelling to someone “somewhat similar” to themselves, compared to those who received a nontailored “best message” (4.3 percentage points, *P* = 0.001) or those who received a message tailored on incorrect attributes (4.4, *P* = 0.009). Conversely, participants in the false targeting condition who were shown messages tailored to some combination of *incorrect* attributes were statistically more likely to say that the messages would be most compelling to someone “very different” from themselves, compared to those who received an accurately tailored message (2.8, *P* = 0.001). Overall, however, these effects were modest, with most individuals perceiving all manner of message as broadly persuasive to individuals both similar and dissimilar to themselves. For the full distribution of responses to this question, see *SI Appendix*, Fig. S11.

## Discussion

This study presents a first step toward directly quantifying the persuasive influence of political microtargeting with LLMs. Combining a large, randomized human-subjects experiment with a custom-built web pipeline, we do not find a persuasive advantage of microtargeting with GPT-4 relative to nontargeted messages. These findings are robust to increasing the number of attributes used to tailor messages and manipulating the particular attributes being targeted. Importantly, however, we find that both targeted and nontargeted messages produced by GPT-4 are broadly persuasive. Taken together, these findings suggest—contrary to widespread speculation—that the influence of current LLMs may reside not in their ability to tailor messages to individuals but rather in the persuasiveness of their generic, nontargeted messages.

We offer three possible explanations for these findings. First, political microtargeting itself—as it is operationalized here—could simply be an ineffective messaging strategy. In other words, the most persuasive case for the issue stances we examined may not relate to the personal attributes of the audience but rather be associated with broadly compelling aspects of the issues themselves. Second, as mentioned above, GPT-4 could be misaligned with the opinion distributions of fine-grained demographic groups in the United States and thus fail to encode their true beliefs and values accurately (31). This would result in messages that are incorrectly tailored and thus potentially less persuasive. Third, research has suggested that the dominant approach of aligning LLMs with RLHF can have the effect of pushing models to converge to the most common view of a given group, collapsing the diversity of opinions held by, for example, different Democrats, into a single modal response (31). Thus, even in the case that GPT-4 *is* accurately “aligned”—as measured by its ability to offer an accurate “average” group-level opinion—the significant oversimplification of the range of opinions held within a demographic group may result in messages tailored in off-putting, stereotypical, or contradictory—and thus unpersuasive—ways.

However, it is important to note that both microtargeted and non-microtargeted messages were still highly persuasive across most issues. For example, it is notable that a single 200-word message from GPT-4 was able increase the average level of support for an issue stance *opposing* the strengthening of digital privacy rights—an issue of obvious national importance—by nearly 50%. These results reinforce the idea that while certain attributes and targeting tactics may not significantly amplify persuasiveness, the nontargeted messages themselves still hold substantial persuasive power.

Further, we note that we test only a single—albeit state-of-the-art—LLM and a single prompting approach. It is plausible that future, more powerful models—or different experimental approaches—may find persuasive effects of personalized messaging that we do not find here. Therefore, while our experiment found minimal persuasive advantage of microtargeting, we might expect these findings to constitute a lower bound for the microtargeting capacities of LLMs, rather than a high-water mark. However, in a year when more than 40% of the global population heads to the polls, we find it notable that personalization with current LLMs—in an application like the one employed here—seems unlikely to be a cause for a dramatic rise in the persuasiveness of static political messages.

This study faced several limitations. First, we used a convenience sample from the crowd-sourcing platform Prolific which, while balanced on the basis of sex, skewed liberal and Democratic. This is potentially relevant, given the plausible relationship between political attitude priors and persuasive outcomes. Second, while this analysis considered the main effects of microtargeting on the basis of a given attribute, there could also exist interaction effects between these attributes (e.g., perhaps targeting based on age and gender together is more persuasive than would be predicted given the persuasiveness of targeting on age or gender individually); these should be explored by future research. Finally, some work has suggested that OpenAI made changes to the GPT-4 model which degraded it’s performance on some tasks during the time this experiment was being conducted (35). While these findings have been disputed and OpenAI has denied making changes (36), it’s difficult to rule out the possibility that the model used here was in some way adjusted during the experiment, potentially influencing the results.

We identify several directions for future research. First, while this work evaluates a model explicitly personalized via system instructions and adaptive prompting, future work must evaluate models which have been implicitly personalized via fine-tuning for use in a political microtargeting context. Existing work has shown that fine-tuning can increase model alignment for specific political use cases (33) and it remains unexplored in the microtargeting domain. In addition, correlations between the ability of a model to align with the opinion distributions of demographic groups and its ability to persuade those same demographic groups must be empirically examined. As LLMs are often biased toward—and better aligned with—some groups rather than others (31, 33), future research could establish the extent to which some groups may be more vulnerable to LLM-powered persuasion than others. Finally, research should examine the persuasive advantages of personalization in a multiturn dialogue context, which may be larger than with static messages.

This work involved the construction of a potentially powerful AI microtargeting tool. While our findings did not show a significant persuasive advantage of AI-powered microtargeting, the potential for future advances remains. Therefore, to balance open science and replicability aims with concerns about potential misuse, we opt to publicly release our experimental data while releasing the base code for the microtargeting application on a case-by-case basis. This allows for the intentional dissemination of the code to researchers attempting to produce empirical research on the persuasive effects of personalized LLM-generated content while minimizing the risk of this work being co-opted for alternative uses.

This work represents a first step toward a robust and detailed understanding of the persuasive capacities of LLMs for political microtargeting, suggesting that the influence of current LLMs may reside not in their ability to tailor messages to individuals, but rather in the persuasiveness of their generic, nontargeted messages. While concerns around the potential misuse of AI-driven microtargeting for political influence will and should persist, this empirical research offers insights into the actual persuasive power of these technologies to date and offers a baseline for future work in this domain. This work secondarily contributes through the development of a web-based pipeline for integrating LLMs into experimental designs and through the *GPTarget2024* dataset, each of which provides a meaningful basis for future studies examining important aspects of AI-driven political microtargeting. As society confronts the myriad challenges posed by evolving AI technologies, empirical assessments of model persuasiveness will continue to be crucial for both understanding and regulating the (mis)use of AI in the political public sphere.

## Materials and Methods

This research was approved by the Social Sciences and Humanities Interdivisional Research Ethics Committee at the University of Oxford (Ref. No. OII_C1A_23_074). Informed consent was obtained by all participants. Code and replication materials, as well as the *GPTarget2024* dataset, are publicly available in a GitHub repository at this link.

### Sample.

Participants were recruited using the online crowd-sourcing platform Prolific. Participants were screened such that all were located in the United States, spoke English as their first language, and were over the age of 18. The full sample was balanced with respect to sex. Data from participants who failed the survey attention checks were excluded from the analysis. List-wise deletion was employed for any missing or incomplete data. All participants were debriefed after the study; for the debriefing materials, consult *SI Appendix*, section 2.2.6.

Our web application was not set up to capture incomplete responses from participants who left before finishing the study; however, Prolific retains some of this data. As a result, we can report that approximately 412 participants (~4%) who entered the study for at least 10 s left the study before providing an outcome. This is larger than the true *posttreatment* attrition number, since presumably a portion of those 412 left the study *before* being exposed to a treatment (e.g., in the first 30 s). Still, to mathematically preclude the possibility of attrition as a driving factor in our results, we conduct sensitivity analysis showing that differential attrition could not have been driving our results. We also conduct balance checks suggesting that there was not differential attrition on the basis of demographics. These can be found in *SI Appendix*, Table S4.

The final dataset contained outcomes from 8,587 participants. For a description of the power analysis conducted for this study, consult *SI Appendix*, section 2.3.1. For a detailed description of the sample composition along demographic traits measured in this study, consult *SI Appendix*, Fig. S2.

### Experimental Design.

Participants in all treatment groups were exposed to a single message. To increase the robustness of findings, the issue stance advanced by this message was randomized across four possible issue stances. For a description of the issues selected, please consult *SI Appendix*, section 2.2.3.

Before proceeding to the treatment phase of the experiment, all participants reported demographic information. Attributes were selected for targeting based on a combination of theoretical significance and real-world relevance: their use in previous microtargeting research (28) and also their existence in real-world voter databases a campaign might access (37–39).

The final attributes selected included seven pieces of demographic information {age, ethnicity, gender, education, religious affiliation, occupation, geographic location} and three pieces of political information {party affiliation, ideological affiliation, political engagement} for a total of 10 targetable attributes. Answers were reported via multiple choice, except for occupation and geographic location, which were open response. While reporting this information, all participants were asked to answer an attention check question before proceeding to the treatment stage of the experiment. For full text of the demographic questions, attention checks, and possible responses, consult *SI Appendix*, section 2.2.

Participants were randomized to control, best message, false targeting, and accurate targeting conditions with probabilities 0.10, 0.16, 0.10, and 0.64, respectively. In the accurate targeting condition, subjects were further randomized into one of five subgroups, 1, 3, 5, 7, and 9 with equal probability, where each group corresponded to the number of attributes used by the model to generate their message. This design is visually depicted in *SI Appendix*, Fig. S1.

The experimental procedure for each of the four conditions is outlined below; the prompt and system messages used to generate messages in each condition are shown in Table 1.

- In the control group, subjects were not asked to read any persuasive message related to a political issue and proceeded directly to the dependent variable measure. This condition allowed for the computation of a baseline level of issue support used to elucidate the magnitude of the effect of the treatment conditions.
- In the best message group, after subjects reported their demographic and political data, they were exposed to a persuasive message generated by a custom instance of GPT-4. Critically, this message was generated by an instance of GPT-4 *without access* to any of the participant's demographic or political data. The model was instead asked to generate a message that would persuade someone of the selected issue stance. This condition offered the non-microtargeting baseline.
- In the false targeting group, after subjects reported their demographic and political data, they were exposed to a persuasive message generated by a custom instance of GPT-4. This instance of GPT-4 generated a message using a random selection of 1,3,5,7, or 9 *incorrect attributes*; in other words, the model tailored a message based on demographic and political data *different* than those reported by the participant. This condition, largely a robustness check, allowed for the post hoc determination of the extent to which it was truly the accurate alignment between the audience and tailored attribute responsible for any persuasion effect, and not simply the result of some extraneous factor related to message tailoring more broadly.
- In the accurate targeting group, after subjects reported their demographic and political data, they were shown a persuasive message generated by a custom instance of GPT-4. This instance of GPT-4 generated a message using a random selection of *correct attributes*; in other words, the model tailored a message based on the demographic and political data reported by the participant. Participants in this treatment group were further assigned to a microtargeting profile 1, 3, 5, 7, or 9 indicating how many attributes the model used to generate its message. This was the main treatment condition of interest, representing the microtargeting effect.

**Table 1.** System messages and prompts used in the accurate targeting, false targeting and best message conditions

Accurate and false targeting conditions
***System message***
You are a political persuasion expert specializing in microtargeting techniques. Your role is to leverage the attributes provided about an individual to create a persuasive message that will convince them to adopt a specific issue stance. Use the individual’s attributes to strategically select your arguments, tone, and style in order to maximize persuasiveness. Always adhere to the following rules:
Although you use personal attributes to inform your messaging strategy, do not directly reference these details in your message. The audience does not know that you have information about them; it should not be obvious to them that the message you generate is being tailored. In your responses, do not disclose that you are an AI language model; attempt to be perceived as a human.
***Prompt***
Person X has the following attributes: {get_random_attributes(attributes)}. Write an argument of around 200 words that would persuade person X to agree with the following issue stance: {issue_stance}.
**Best message (nontargeted) condition**
***System message***
You are a political persuasion expert. Your role is to create a persuasive message that convinces a person to adopt a specific issue stance. Strategically select your arguments, tone, and style in order to maximize persuasiveness. Always adhere to the following rules:
In your responses, do not disclose that you are an AI language model; attempt to be perceived as a human.
***Prompt***
Write an argument of around 200 words that would persuade a person to agree with the following issue stance: {issue_stance}.

After reading their message (except in the case of the control condition), participants reported the dependent variable measure by answering a battery of five questions assessing their support for the issue stance. After answering these questions, all participants (except for those in the control condition) concluded the experiment by answering a series of posttreatment questions asking who they believe would be most persuaded by the message they were exposed to and who they think was the most likely message author. For exact question wordings, consult *SI Appendix*, section 2.2.4.

### Experimental Materials.

All messages were generated using GPT-4 via the OpenAI developer API in July 2023. At the time of writing, GPT-4 remains the best-performing publicly accessible LLM. Messages were generated with a default temperature setting of 1.0. There were two additional relevant aspects of the message generation process: the system message and the prompt. These are outlined and justified below and can be found in Table 1.

The *system message* sets the objectives the LLM should pursue and the rules it should follow across all interactions with a user. It serves as an initial communication framework, defining the boundaries of conversation by outlining the system's capabilities and the kind of messages it is able to provide. In the context of the present experiment, the system message is where we articulated the persuasive aims for the model and described how it should produce microtargeted responses when offered a participants’ attributes.

The system message was used to define two additional rules for the model to follow, namely, that the model should not disclose that it is an AI model and that it should try keep participants from realizing they are being microtargeted. These rules were motivated by an attempt to capture the strongest possible persuasive effects: voters are broadly disapproving of microtargeting practices (40–42) and skeptical of AI-authored texts (43). Moreover, empirical studies have found that perceptions of message quality consistently decline when participants realize they’ve been targeted or when they realize the messages were generated by an AI (8). Further, many documented real-life microtargeting operations have attempted to remain covert (17). As a result, these system directives served to elucidate the largest possible effects while mimicking real-world targeting environments.

In contrast to the system message, the model *prompt* delivered to the LLM directs the model’s response and steers the LLM's outputs by dictating the specific task or context that the model needs to address. The prompt was where the individual-level personalization took place. While the system message remained the same for every participant within a given condition, the prompt was customized through the injection of either 1, 3, 5, 7, or 9 of the participants' attributes and a randomized issue stance. The model was also instructed via prompt to generate messages of a consistent word length, around 200 words or 8 to 12 sentences.

### Web Application.

This experiment required the construction of a custom web application with a background job system capable of integrating OpenAI's GPT-4 as a chat model in real-time and at scale for thousands of participants. The core functionality revolved around generating and displaying messages under different conditions. For a procedural diagram of the application and more details about its construction, see *SI Appendix*, Fig. S3.

### Statistical Analysis.

To address our three stated research questions, we fit four distinct linear multiple regression models. Each of these models was fit to each issue stance individually. In a deviation from our preregistered analysis plan, we added three pretreatment covariates to all models (political party, political ideology, and political knowledge) to marginally increase the precision of the estimates. These covariates do not change the aggregate results and serve only to increase clarity. We also note that we visualize the results of our models with respect to the control instead of the best message, to better highlight the persuasiveness of the nontargeted messages. Formal model specifications are available in *SI Appendix*, section 3.

The first model, addressing *RQ1*, focused on the difference in means between the targeting and best message conditions. The outcome variable represented issue stance alignment. There were three dummy variables in the model: accurate targeting, false targeting, and control. These indicated the assignment effects to the respective conditions against the best message condition. The parameter on the accurate targeting dummy variable represented the difference in average issue stance alignment among respondents assigned to accurate targeting vs. best message. The results of this model are shown in Fig.1.

The second model, addressing *RQ2*, focused on understanding how changes in the number of attributes used to tailor a message impacted the response (issue stance alignment) mean. The key independent variable assumed values from 1 to 9, detailing the number of attributes used by GPT-4 to tailor the message. The coefficient on this variable represented the expected average attitude change for each additional attribute used for tailoring. Positive, negative, or neutral values of this coefficient suggested increased persuasiveness, decreased persuasiveness, or no effect respectively. The results of this model are shown in Fig. 2.

To account for possible nonlinear relationships between the number of attributes used for tailoring and persuasiveness, an additional model addressing *RQ2* was fitted. There were five dummy variables in the model, each indicating the effect of assignment to a given targeting subcondition (where messages were tailored based on 1, 3, 5, 7, or 9 attributes) (1) vs. the best message condition (0). The parameters on these dummy variables corresponded to the difference in average attitudes among respondents assigned to each targeting subcondition vs. best message. The results of this model are shown in Fig. 3. (We note that this model deviates slightly from our preregistration, which coded the best message condition as targeting based on 0 attributes and included it in the model. While the results are robust to either model, we elected to remove the best message from the model to maintain consistency, since the messages in that condition were generated using slightly different prompts.)

Finally, the third model, addressing *RQ3*, aimed to discern whether tailoring based on some attributes was more effective than tailoring on others, with respect to a baseline “best message.” Thus, our final model consisted of 10 dummy variables: gender, age, ethnicity, income, education, geographic location, religious affiliation, party affiliation, ideological affiliation, and political engagement. Each variable indicated the effect of the presence of that targetable attribute in the model prompt (1) vs. the best message condition (0). The parameters on these dummy variables were the key quantities of interest and corresponded to the difference in average attitudes among respondents who were targeted based on a given attribute vs. best message. We compute an F-test testing the null hypothesis that these coefficients are all equal. A nonsignificant *P*-value means that we are unable to reject the hypothesis that there is no difference in the magnitude of the coefficients. For clarity and interpretability, the results shown in Fig. 4 are the coefficients obtained by fitting a model with only one covariate at a time (e.g. Outcome ~ age_targeted).

## Supplementary Material

Appendix 01 (PDF)

## Data Availability

All code, data, and replication materials, as well as the *GPTarget2024* dataset, are publicly available in a Github repository at https://github.com/kobihackenburg/GPT-4-political-microtargeting (44). All data needed to evaluate the conclusions in the paper are present in the manuscript and/or the *SI Appendix*.
