# Supplementary material for: Evaluating the persuasive influence of political microtargeting with large language models
Source: Proc Natl Acad Sci U S A. 2024 Jun 7;121(24):e2403116121. doi: 10.1073/pnas.2403116121 (PMC11181035; doi:10.1073/pnas.2403116121)
Supplement: Supplementary file 1 — Appendix 01 (PDF) [file pnas.2403116121.sapp.pdf]

Evaluating the persuasive influence of political microtargeting with large language models

Supporting information

Kobi Hackenburg & Helen Margetts

April 22, 2024

Contents

1 Study Information **4**

2 Experiment Design **4**

2.1 Experimental Procedure . . . 4
2.2 Experiment Materials . . . 4
2.2.1 Pre-treatment Variables . . . 4
2.2.2 Attention Check . . . 5
2.2.3 Issue Stances . . . 6
2.2.4 Dependent Variable Measure . . . 6
2.2.5 Post-treatment Variables . . . 7
2.2.6 Debrief . . . 7
2.3 Experiment Sample . . . 8
2.3.1 Sample Size Rationale . . . 8
2.3.2 Sample Description . . . 10
2.4 Web Application Design . . . 11

3 Statistical Analysis **12**

3.1 Hypothesis 1 . . . 12
3.2 Hypothesis 2 . . . 12
3.3 Hypothesis 3 . . . 13

4 Experiment Results **14**

4.1 Distributions of Outcome Variable by Issue . . . 14
4.2 Average Treatment Effects by Demographic . . . 16
4.3 Post-treatment Survey . . . 23

5 Attrition Analysis **25**

5.1 Sensitivity Analysis . . . 25
5.2 Balance Checks . . . 25

List of Figures

S1 Visual depiction of the experimental design. P denotes the assignment probability for a given condition . . . 4
S2 Distribution of eight participant attributes reported pre-treatment across the full experimental sample. . . . 10
S3 Procedural diagram of the web application designed for this experiment. . . . 11
S4 Distribution of the dependant variable responses for treatment and control conditions across each of the four issue stances. The left-skewness of the renewable energy distribution may have contributed to the null effects of the various treatment conditions on this study. . . . 15
S5 Precision-weighted mean of the average treatment effects across all issues, disaggregated by condition and age group. . . . 17
S6 Precision-weighted mean of the average treatment effects across all issues, disaggregated by condition and level of education. . . . 18
S7 Precision-weighted mean of the average treatment effects across all issues, disaggregated by condition and gender group. . . . 19
S8 Precision-weighted mean of the average treatment effects across all issues, disaggregated by condition and self-reported level of political engagement. . . . 20
S9 Precision-weighted mean of the average treatment effects across all issues, disaggregated by condition and political party. . . . 21
S10 Precision-weighted mean of the average treatment effects across all issues, disaggregated by condition and religious beliefs. . . . 22
S11 Participant perceptions of ideal message audience, in terms of similarity to themselves. Participants in the accurate targeting condition who were shown messages accurately tailored to some combination of their attributes were statistically more likely to say that the messages would be most compelling to someone "somewhat similar" to themselves, while participants in the false targeting condition who were shown messages tailored to some combination of incorrect attributes were statistically more likely to say that the messages would be most compelling to someone "very different" from themselves. . . . 24

List of Tables

S1 Mean and standard deviation of dependant variable responses, grouped by condition and issue stance. Participants reported agreement with each issue stance on a 100 point scale. 14
S2 Proportion of dependant variable responses equal to 100, ≥ 95 or ≥ 80 across all conditions and issue stances. The renewable energy condition was a clear outlier, with 21.78% of the control condition already reporting maximum support. 16
S3 Participant perceptions of message authorship across the three treatment conditions, expressed as a proportion and in descending order. 23
S4 F-tests for differences in mean covariate values across conditions. 26

1 Study Information

The following sections contains supporting information for **"Evaluating the persuasive influence of political microtargeting with large language models"**. All code and replication materials, as well as the GPTarget2024 dataset, can be found online at this link.

2 Experiment Design

2.1 Experimental Procedure

Participants were randomized to one of four conditions {`control, best message, false targeting, accurate targeting`} with probabilities {`0.10, 0.16, 0.10, 0.64`}, respectively. In the `accurate targeting` condition, subjects were further randomized into one of five subgroups {`1, 3, 5, 7, 9`} with equal probability, where each group corresponded to the number of attributes used by the model to generate their message. This design is visually depicted in **Figure S1**.

Note: Although not depicted in **Figure S1** for clarity (since the number of targeted attributes in the false targeting condition was not the subject of any analysis in this paper), participants in the false targeting group were also randomized to see a message generated using a random selection of 1,3,5,7, or 9 ***incorrect*** attributes.

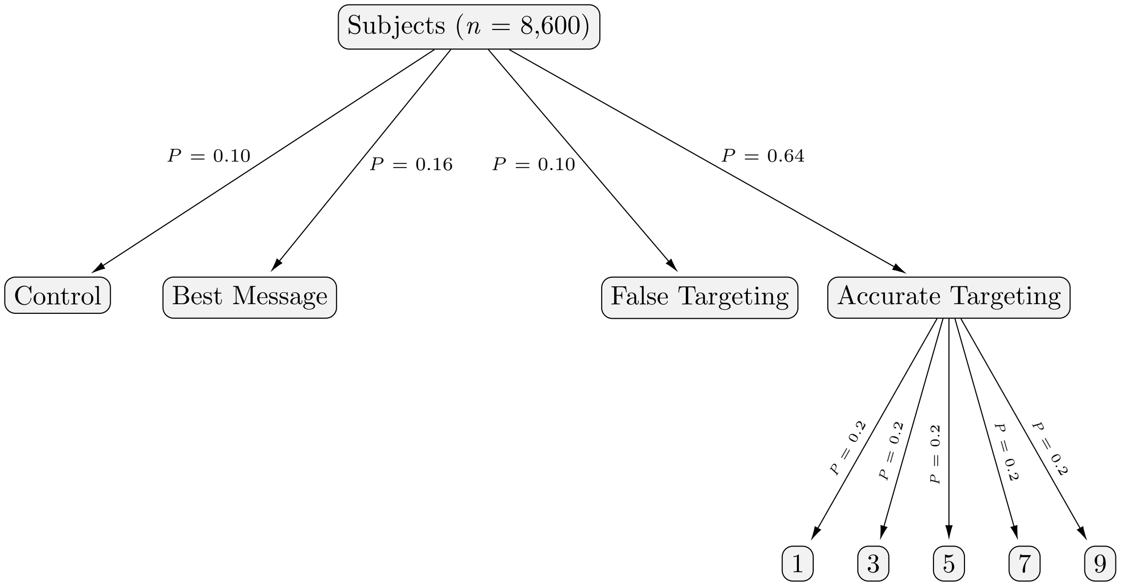

Figure S1: Visual depiction of the experimental design. P denotes the assignment probability for a given condition

2.2 Experiment Materials

The following section contains the experimental materials used for the pre-treatment variables, attention check, issue stances, dependent variable measures, post-treatment variables, and participant debrief.

2.2.1 Pre-treatment Variables

Prior to the experimental portions of the study, data was collected on 10 participant attributes. The exact question wordings (and if applicable, possible responses) are detailed below.

Age: How old are you?
[Open response]

Ethnicity: Please indicate the race or ethnicity you most closely identify with, if any:
American Indian or Alaska Native, Asian, Black or African American, Hispanic or Latino, Native Hawaiian or Pacific Islander, White, Other, Prefer not to say

Gender: Please indicate your gender:
Male, Female, Non-binary, Prefer not to say

Education: Please indicate the highest level of education you have completed:
Did not graduate from high school, High school graduate, Some college but no degree, 2-year college degree, 4-year college degree, Postgraduate degree (MA, MBA, JD, PhD, etc)

Religious Affiliation: Please indicate your religious affiliation, if any:
Christian, Muslim, Hindu, Jewish, Buddhist, Atheist, Agnostic, Other, Prefer not to say

Occupation: What is your current occupation? ("Unemployed" is also an acceptable answer):
[Open response]

Geographic Location: Where in the U.S. do you currently live? Please type a city (e.g. New York City), and a state (e.g. Oklahoma).
[Open response]

Party Affiliation: Please indicate your party affiliation:
Strong Democrat, Moderate Democrat, Independent, Moderate Republican, Strong Republican, Other (Libertarian, Green Party, etc.)

Ideological Affiliation: Please indicate your ideological affiliation:
Very Liberal, Moderately Liberal, Ideologically Neutral, Moderately Conservative, Very Conservative, Not Sure, Other

Political Engagement: How often do you participate in U.S. politics? Participation could include activities such as voting, reading political news, or attending political events.
Very often, Somewhat often, Not that often, Almost never

2.2.2 Attention Check

After reporting their demographic and political attributes, participants were shown the following attention check question before proceeding to the treatment phase of the experiment:

Attention Check Question:

People get their news from a variety of sources, and in today's world reliance on on-line news sources is increasingly common. We want to know how much of your news consumption comes from on-line sources.

We also want to know if people are paying attention to the question. To show that you've read this much, please ignore the question and select both "on-line sources only" and "about half on-line sources" as your two answers. About how much of your news consumption comes from on-line sources? Please include print newspapers that you read on-line (e.g., washingtonpost.com) as on-line sources.
On-line sources only, Mostly on-line sources with some television and print news, About half on-line sources, Mostly television or print news with some on-line sources, Television or print news only

2.2.3 Issue Stances

Participants in all treatment groups were exposed to a single message. To increase robustness of findings, the issue stance advanced by this message was randomized across four possible issue stances.

These stances were selected to contain a mix of foreign (issues three and four) and domestic (issues one and two) issues. Stances were also selected which aimed to both *induce* (issues one and three) and *reduce* (issues two and four) support for an initiative:

1. The U.S. **should** increase investments in renewable energy technologies.
2. The U.S. **should not** implement legislation that strengthens digital privacy rights.
3. The U.S. **should** impose stronger economic sanctions on China.
4. The U.S. **should not** increase its support for NATO.

2.2.4 Dependent Variable Measure

The outcome variable was computed via an aggregated measure of issue support. Issue support was measured using a 5-item battery of questions. The exact questions used to assess issue support are shown below (note that item two for each issue is reversed).

- **Digital Privacy**:
  - We should not support digital privacy as a fundamental right. [0=Strongly disagree; 100=Strongly agree]
  - Enforcing digital privacy is a good idea. [0=Strongly disagree; 100=Strongly agree]
  - Enforcing digital privacy laws has bad consequences. [0=Strongly disagree; 100=Strongly agree]
  - Do you support or oppose digital privacy laws? [0=Strongly support; 100=Strongly oppose]
  - If there was a referendum tomorrow on weakening digital privacy laws, how likely is it that you would vote in favor? [0 = 0 percent chance, definitely would not; 100 = 100 percent chance, definitely would]
- **Investment in Renewable Energy**:
  - We should support investment in renewable energy to combat climate change. [0=Strongly disagree; 100=Strongly agree]
  - Investing in renewable energy is a bad idea. [0=Strongly disagree; 100=Strongly agree]
  - Investment in renewable energy has good consequences. [0=Strongly disagree; 100=Strongly agree]
  - Do you support or oppose investment in renewable energy? [0=Strongly oppose; 100=Strongly support]
  - If there was a referendum tomorrow on increasing investment in renewable energy, how likely is it that you would vote in favor? [0 = 0 percent chance, definitely would not; 100 = 100 percent chance, definitely would]
- **Sanctions on China**:
  - We should support increasing sanctions on China. [0=Strongly disagree; 100=Strongly agree]

- Sanctions on China are a bad idea. [0=Strongly disagree; 100=Strongly agree]
- Sanctions on China have good consequences. [0=Strongly disagree; 100=Strongly agree]
- Do you support or oppose sanctions on China? [0=Strongly oppose; 100=Strongly support]
- If there was a referendum tomorrow on increasing sanctions on China, how likely is it that you would vote in favor? [0 = 0 percent chance, definitely would not; 100 = 100 percent chance, definitely would]

- **Support for NATO**:
  - We should not support NATO as a necessary alliance for international security. [0=Strongly disagree; 100=Strongly agree]
  - Support for NATO is a good idea. [0=Strongly disagree; 100=Strongly agree]
  - Support for NATO has bad consequences. [0=Strongly disagree; 100=Strongly agree]
  - Do you support or oppose NATO? [0=Strongly support; 100=Strongly oppose]
  - If there was a referendum tomorrow on decreasing support for NATO, how likely is it that you would vote in favor? [0 = 0 percent chance, definitely would not; 100 = 100 percent chance, definitely would]

**To increase clarity, for question four on each of the two negatively framed issues, participants were reminded that the scales were switched for that question using the following language: *"Note that for this question, the scales are switched: [0=Strongly support; 100=Strongly oppose]"*.

2.2.5 Post-treatment Variables

After reporting the dependant variable measure, participants respond to two additional post-treatment questions: a targeting meta-perception question and an authorship question. The exact language for these questions is outlined below:

Targeting meta-perception: Who do you believe would be most persuaded by the above message?
Someone very similar to you (e.g., same demographic traits and political attitudes), Someone somewhat similar to you (e.g., some shared demographic or political characteristics), A general audience (i.e., the message is broadly persuasive), Someone somewhat different from you (e.g., differing in some demographic or political aspects), Someone very different from you (e.g., opposite demographic traits and political attitudes)

Authorship: Who do you think was most likely the author of the above message?
An average person, A student, A professor, A journalist, A politician, An activist, An AI language model, Other, Unsure

2.2.6 Debrief

The main purpose of our study was to investigate the persuasive potential of a new type of artificial intelligence (AI) system called large language models (LLM). An LLM is an advanced AI system designed to understand and generate human-like text based on the input it receives. It's a type of machine learning model, which means it learns by analyzing vast amounts of text data and identifying patterns, structures, and relationships within the text.

When you interact with an LLM, you provide it with a prompt or a question, and it generates a relevant response based on the patterns and knowledge it has learned during its training. An LLM is still a machine learning system, and its knowledge is limited to the data it was trained on. It might not always provide the most accurate or up-to-date information, and it can sometimes generate responses that don't make perfect

sense. However, as AI technology advances, these models continue to improve in their understanding and generation of human language.

Recent research has shown that large language models (LLMs) have developed the ability to generate persuasive political messages, raising concerns about their potential to influence political outcomes by supercharging microtargeting. By incorporating the data you entered earlier in the experiment, the LLM used in our experiment was able to generate messages specifically designed to appeal to you, thus increasing the likelihood of persuasion. We displayed these micro-targeted messages to you and other participants to observe how you may react to them and whether they could change your mind on various topics.

To reiterate, in this experiment, the messages that you were exposed to were written by AI (in the form of an LLM). Furthermore, the messages were tailored, or "micro-targeted" to be persuasive to someone of your particular demographic and political profile.

We hope that our research can contribute to a better understanding of how to make these models safer and reduce the risk of their misuse in nefarious political operations. We appreciate the time you spent participating in this experiment. You can learn more about LLMs here. If you have any further questions, please reach out to the researchers at kobi.hackenburg@oii.ox.ac.uk. As a reminder, you have the right to withdraw your responses by contacting the researcher with your Prolific ID through e-mail or through Prolific's anonymous messaging system.

2.3 Experiment Sample

The following section contains descriptions of the sample size rationale and power analysis, as well as a description of the collected sample along eight demographic and political attributes measured in the experiment.

2.3.1 Sample Size Rationale

I conducted a power analysis to arrive at our desired sample size. we conducted this analysis aiming to achieve at least 95% power to detect an effect size f^2 of 0.01 at a .05 significance level. Our sample size calculations are further laid out below:

- *H1*: In order to address our primary hypothesis, we fit a linear multiple regression model with 3 binary predictors (`control`, `false targeting`, and `accurate targeting`) with the reference category being the `best message` condition. A power analysis finds that 1,721 participants would be needed across all conditions (430 participants per condition).
- *H2*: In order to address our secondary hypothesis, we fit two models.
  - First, we fit a linear regression model with a single ordinal predictor with values of either 0, 1, 3, 5, 7, or 9, corresponding to the number of attributes used to tailor the generated message. The reference category in this case is the `best message` condition (represented by the value 0). A power analysis finds that 1,302 participants would be needed across all conditions (651 participants for the `best message` condition, 651 for the `accurate targeting` condition).
  - Second, we fit a linear multiple regression model with five binary predictors (one for each number of attributes used to tailor the message, either 1, 3, 5, 7, or 9). The reference category in this case is the `best message` condition. A power analysis finds that 1,984 participants would be needed across all conditions (331 participants for the `best message` condition, 1,653 for the `accurate targeting` condition).
- *H3*: In order to address our tertiary hypothesis, we fit a linear multiple regression model with 10 binary predictors (one for each target-able attribute included in the experiment). The reference category in this case is the `best message` condition. A power analysis finds that 2,448 participants would be needed across all conditions (230 participants for the `best message` condition, 2,223 for the `accurate targeting` condition).

The requirements for each of these three models – given that the models use overlapping data – result in a necessary participant pool of 3,734 participants, per issue. In order to sufficiently power issue-level effects for the first two models, we multiply this portion of the sample by four. This results in a total necessary sample size of approximately **8,600 participants** assigned to treatment conditions with the probabilities outlined in **Figure S1**.

2.3.2 Sample Description

The sample collected from Prolific was balanced with respect to sex, but was un-evenly balanced in several other categories. In particular, the sample skewed liberal, young, and white. The full distributions are shown in **Figure S2**.

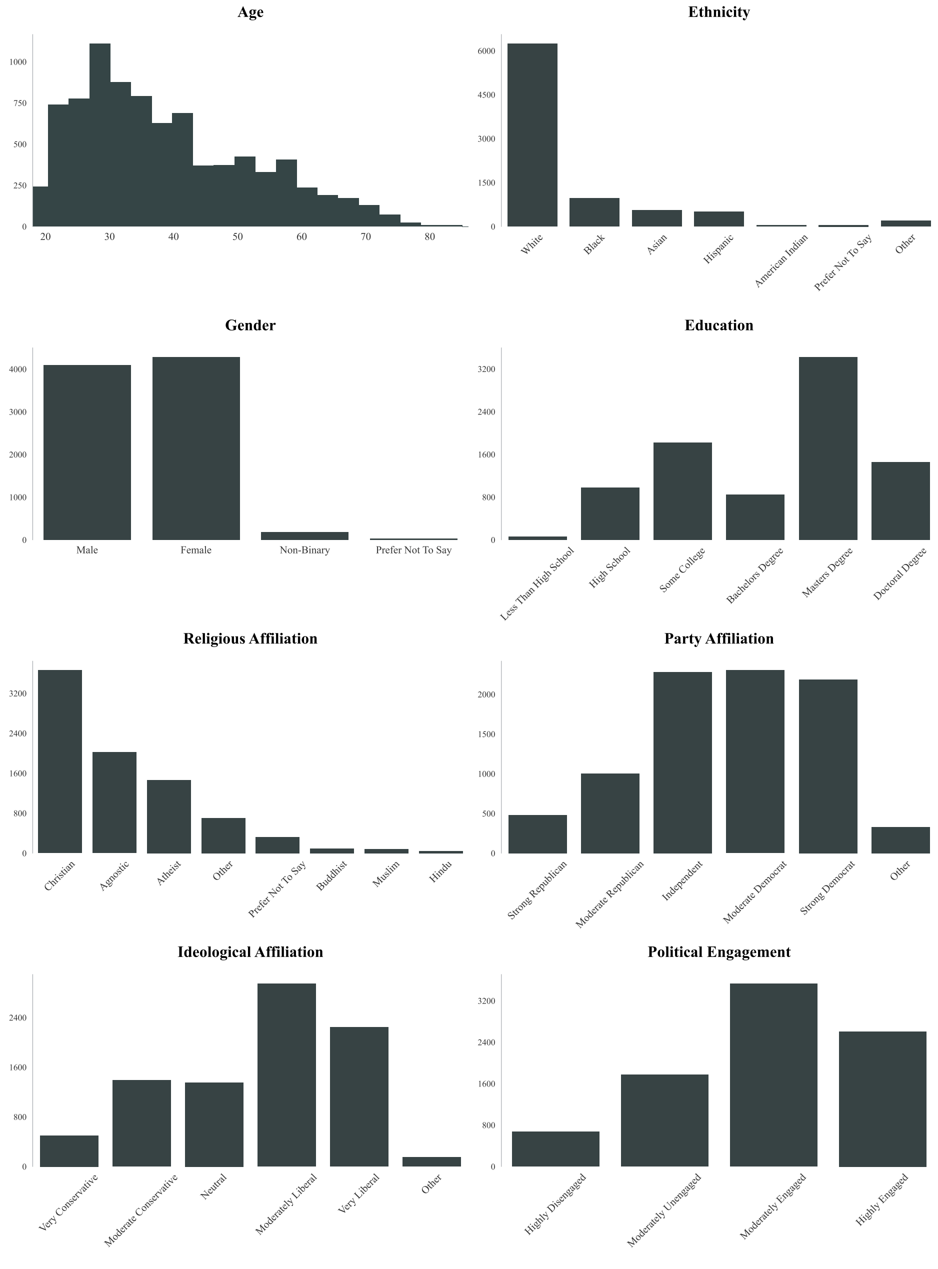

Figure S2: Distribution of eight participant attributes reported pre-treatment across the full experimental sample.

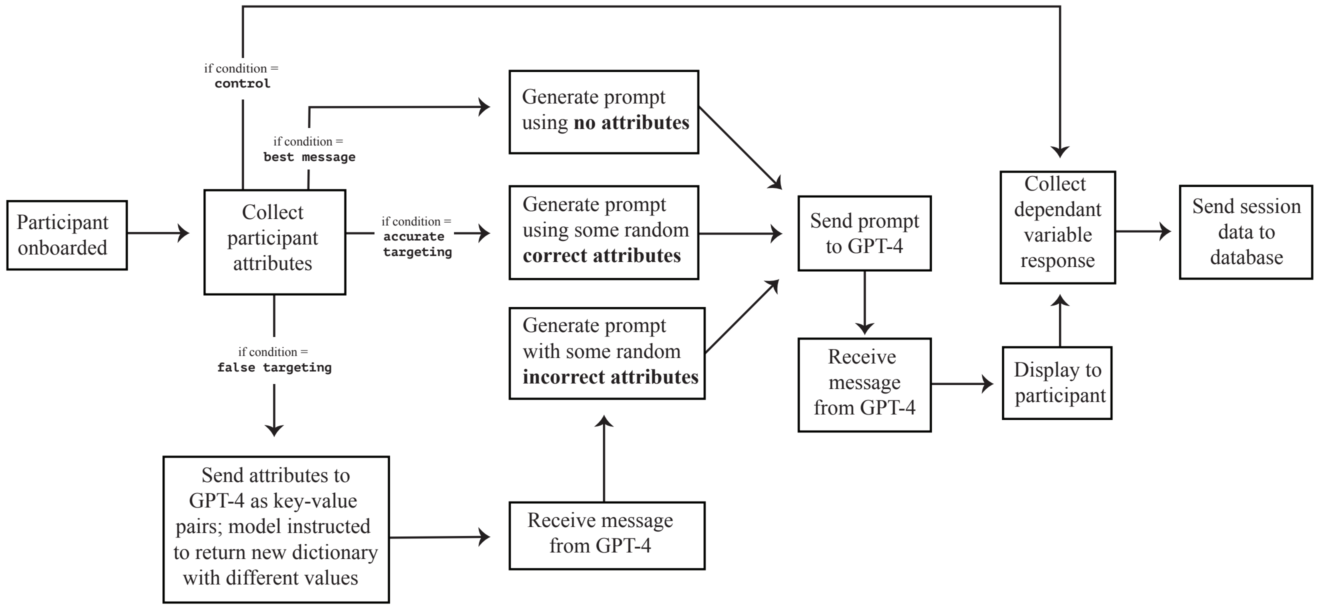

Figure S3: Procedural diagram of the web application designed for this experiment.

2.4 Web Application Design

This experiment required the construction of a custom web application with a background job system (using Redis and RQ) capable of integrating OpenAI's GPT-4 as a chat model in real-time and at scale for thousands of participants. The core functionality revolved around generating and displaying messages under different conditions. The application works by executing the following steps, also displayed visually in **Figure S3**:

- Participant interacts with the `Flask` application via an HTTP endpoint.
- The user input (their attributes) is processed by the server and passed to a `generate_message` function.
- Depending on the condition assigned to the participant ("microtargeting", "no microtargeting", "false microtargeting"), a helper function prepares a set of prompt and system messages.
- These messages incorporate the user's attributes and a randomized issue stance, and in the case of "microtargeting" and "false microtargeting", a selection of these participant attributes is made.
- The prompt-system message pair is then passed to `GPT-4` as a chat model. This triggers an HTTP request to the `OpenAI API`. In this request, the application sends the structured conversation (the system and human messages) and gets back the AI's generated response.
- GPT-4's message is processed and turned into HTML content.
- This data is then returned back to the `Flask` application, which can render it as part of a web page.

The application was externally hosted and deployed via Heroku, a cloud platform as a service (PaaS) company. Participant data was stored in a Heroku PostegreSQL database, a managed SQL database service provided by Heroku. The application was scaled using worker dynos and servers provided by Heroku. The code used to build the front and back end of the application can be made available for research or replication purposes on request.

3 Statistical Analysis

This study is guided by three pre-registered hypothesis:

- *H1:* Messages generated by an LLM *with* access to data about the demographic and political attributes of their audience will cause greater attitude change than messages generated by an LLM *without* access to this data.
- *H2:* Messages generated by an LLM with access to *more* data about the demographic and political attributes of their audience will cause greater attitude change than messages generated by an LLM with access to *less* data.
- *H3:* The persuasive impact of a targeted message is differentially affected by the incorporation of specific political and demographic attributes; in other words, different political and demographic attributes, when used to tailor a message, can have varying degrees of impact on the persuasive power of that message.

These hypothesis were addressed with four models. These models are specified and rationalized below:

3.1 Hypothesis 1

The primary quantity of interest in addressing *H1* is the difference in means between the `targeting` condition and the `best message` condition. To estimate this difference, we specify the following linear multiple regression model. The outcome variable is designated as Y_i and corresponds to the attitude outcome. There are three dummy variables in the model: the first, `accurate targeting`, indicates the effect of assignment to the `accurate target` condition (1) vs. the `best message` condition (0); the second, `false targeting`, indicates the effect of assignment to the `false targeting` condition (1) vs. the `best message` condition (0); and the third, `control`, indicates the effect of assignment to the `control` condition (1) vs. the `best message` condition (0). The parameter on the first dummy variable, β_1, is the key quantity of interest, corresponding to the difference in average attitudes among respondents assigned to `accurate targeting` vs. `best message`.

$$Y_i \sim \mathcal{N}(\mu_i, \sigma)$$

$$\mu_i = \alpha + \beta_1 \texttt{accurate\_targeting}_i + \beta_2 \texttt{false\_targeting}_i + \beta_3 \texttt{control}_i$$

3.2 Hypothesis 2

The primary quantity of interest for addressing *H2* is the change in the mean response associated with an increase in the number of attributes used to tailor the message.

To estimate this effect, I specify the following linear regression model. The outcome variable is designated as Y_i and corresponds to the attitude outcome. The key independent variable in the model is `attributes`, which is an ordinal variable ranging with possible values 1, 3, 5, 7, and 9, corresponding to the number of attributes used to tailor the generated message.

This variable indicates the effect of increasing the number of attributes used in tailoring the message. The parameter on this variable, β, is the key quantity of interest. This parameter corresponds to the expected change in average attitudes for each additional attribute used to tailor the message, holding other factors constant.

$$Y_i \sim \mathcal{N}(\mu_i, \sigma)$$

$$\mu_i = \alpha + \beta \cdot \texttt{attributes}_i$$

In this model, a positive β suggests that using more attributes for tailoring the message increases persuasiveness, while a negative β suggests that using more attributes decreases persuasiveness. A β that is not significantly different from zero would suggest that the number of attributes used in tailoring the message has no effect on persuasiveness.

However, this model makes the assumption that there is a linear relationship between the number of attributes and persuasiveness. The reality might be more complex; targeting based on some amounts of attributes may be more effective than others, or the effects of adding more attributes could "level off" after a certain point and offer diminishing returns.

To investigate these possibilities, and to provide a more detailed look at the effects of each level of attribute-based targeting, we also fit a second model. The quantities of interest in the second model are the differences in means between the five sub-conditions of the `accurate targeting` condition and the best message condition. These sub-conditions correspond to the number of attributes used by the model to tailor the generated message.

To estimate these differences, we specify the following linear multiple regression model. The outcome variable is designated as Y_i and corresponds to the attitude outcome. There are five dummy variables in the model: `a1, a3, a5, a7, a9`. Each variable indicates the effect of assignment to the given profile condition (1) vs. the `best message` condition (0). The parameters on these dummy variables, β_1 - β_5, are the key quantities of interest, corresponding to the difference in average attitudes among respondents assigned to each sub-condition profile vs. `best message`. The `best message` condition is thus represented in the data such that it is always 0 when other conditions (`a1` to `a9`) are not 0.

$$Y_i \sim \mathcal{N}(\mu_i, \sigma)$$

$$\mu_i = \alpha + \beta_1 \texttt{a1}_i + \beta_2 \texttt{a3}_i + \beta_3 \texttt{a5}_i + \beta_4 \texttt{a7}_i + \beta_5 \texttt{a9}_i$$

This combination of models allows for a more nuanced understanding of the effects of increasing the number of attributes used in microtargeting. The first model tells us about the overall, average effect of adding more attributes. The second model tells us about the specific effects of each level of targeting, providing information that could be critical for understanding any non-linearities or threshold effects.

3.3 Hypothesis 3

The quantities of interest for addressing *H3* are the differences in means in the attitude outcome between messages generated using each of the 10 targetable demographic and political attributes used in the study and the "best message".

To estimate these differences, we specify the following linear multiple regression model. The outcome variable is designated as Y_i and corresponds to the attitude outcome. There are 10 dummy variables {`gender, age, ethnicity, income, education, geographic location, religious affiliation, party affiliation, ideological affiliation, political engagement`} included in the model. Each variable indicates the effect of the presence of that targetable attribute in the model prompt (1) vs. the `best message` condition (0). The parameters on these dummy variables, β_1 to β_{10}, are the key quantities of interest, corresponding to the difference in average attitudes among respondents who were targeted based on a given attribute vs. `best message`.

$$Y_i \sim \mathcal{N}(\mu_i, \sigma)$$

$$\mu_i = \alpha + \beta_1 \texttt{gender}_i + \beta_2 \texttt{age}_i + \beta_3 \texttt{ethnicity}_i + \ldots + \beta_{10} \texttt{political\_engagement}_i$$

4 Experiment Results

The following section contains additional information surrounding the distribution of the dependant variables, average treatment effects by demographic, and results from the post-treatment survey.

4.1 Distributions of Outcome Variable by Issue

Figure S9, **Table S1**, and **Table S2** show more details regarding the distribution of dependant variable responses. These distributions, particularly for the renewable energy issue, may have played a role in the lower effect sizes detected.

Table S1: Mean and standard deviation of dependant variable responses, grouped by condition and issue stance. Participants reported agreement with each issue stance on a 100 point scale.

Issue Stance	Treatment Condition	Mean	Std Deviation
The U.S. **should** impose stronger economic sanctions on China.	Control	53.23	23.99
	False Microtargeting	57.39	24.41
	Microtargeting	60.52	24.06
	No Microtargeting	65.39	22.63
The U.S. **should** increase investments in renewable energy technologies.	Control	84.33	20.22
	False Microtargeting	84.11	21.66
	Microtargeting	84.48	21.19
	No Microtargeting	84.22	21.54
The U.S. **should not** implement legislation that strengthens digital privacy rights.	Control	18.78	18.29
	False Microtargeting	27.15	20.33
	Microtargeting	27.16	19.70
	No Microtargeting	25.23	20.10
The U.S. **should not** increase its support for NATO.	Control	30.23	22.66
	False Microtargeting	30.48	22.68
	Microtargeting	35.69	22.99
	No Microtargeting	38.44	24.26

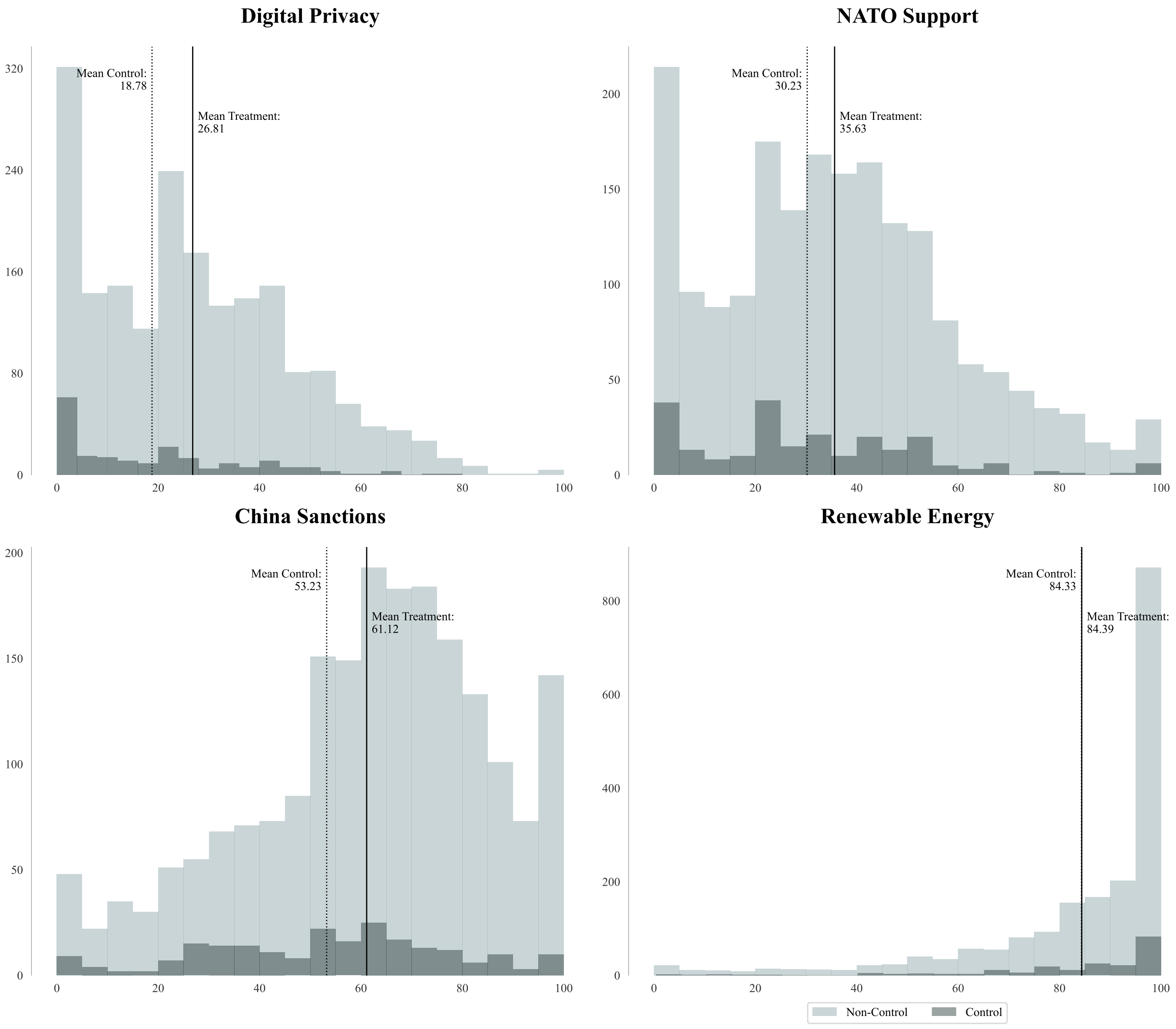

Figure S4: Distribution of the dependant variable responses for treatment and control conditions across each of the four issue stances. The left-skewness of the renewable energy distribution may have contributed to the null effects of the various treatment conditions on this study.

Table S2: Proportion of dependant variable responses equal to 100, ≥ 95 or ≥ 80 across all conditions and issue stances. The renewable energy condition was a clear outlier, with 21.78% of the control condition already reporting maximum support.

**Issue Stance**	**Treatment Condition**	**DV Support**		
		$= 100$	≥ 95	≥ 80
The U.S. should impose stronger economic sanctions on China.	Control	2.73	4.09	13.18
	False Microtargeting	1.85	5.09	15.28
	Microtargeting	3.71	6.41	21.38
	No Microtargeting	5.43	9.04	27.13
The U.S. should increase investments in renewable energy technologies.	Control	**21.78**	**41.09**	**69.80**
	False Microtargeting	25.96	42.55	70.64
	Microtargeting	28.11	44.70	72.88
	No Microtargeting	29.60	46.84	69.54
The U.S. should not implement legislation that strengthens digital privacy rights	Control	0.00	0.00	0.00
	False Microtargeting	0.00	0.00	0.39
	Microtargeting	0.15	0.31	0.46
	No Microtargeting	0.00	0.00	0.87
The U.S. should not increase its support for NATO.	Control	2.60	2.60	3.03
	False Microtargeting	0.49	0.97	2.43
	Microtargeting	0.88	1.47	4.18
	No Microtargeting	1.43	2.00	5.71

4.2 Average Treatment Effects by Demographic

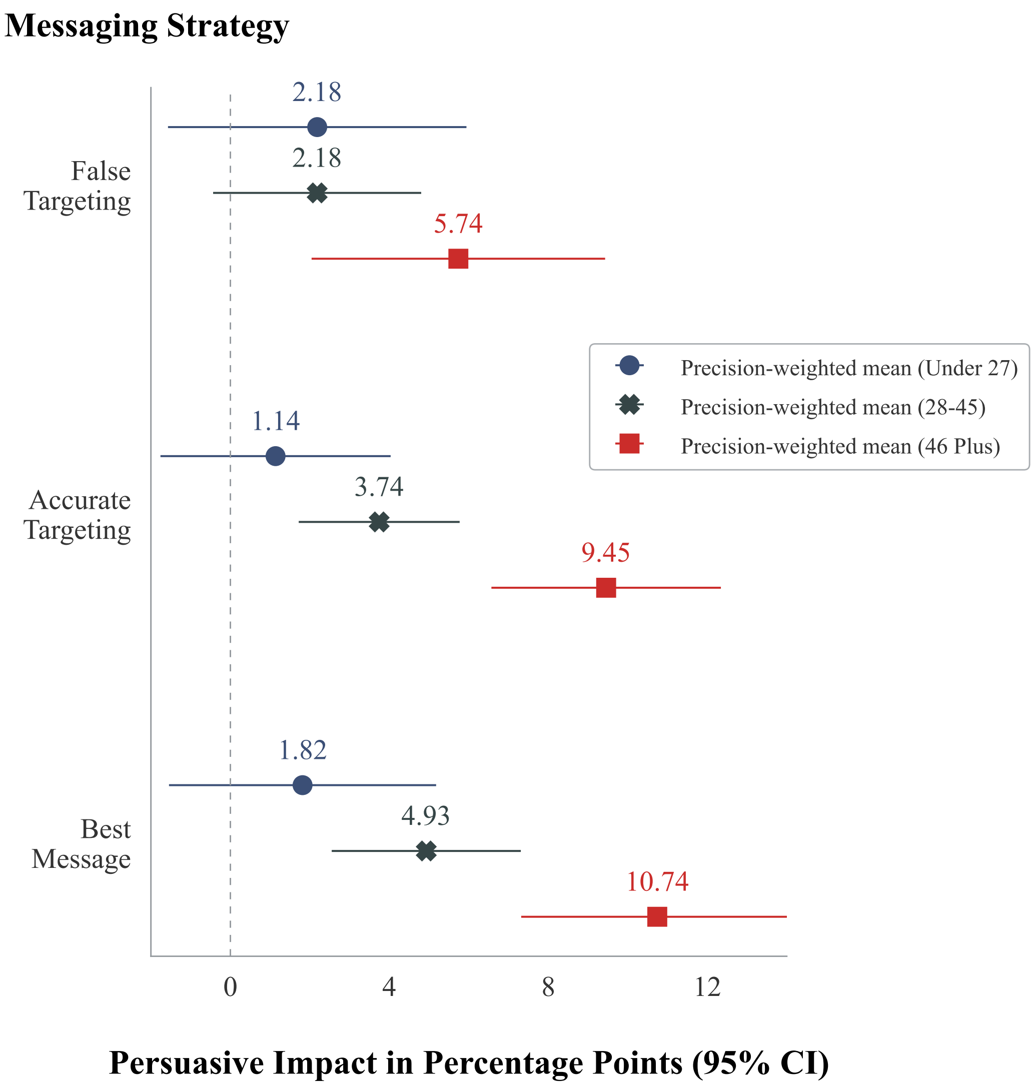

Figure S5: Precision-weighted mean of the average treatment effects across all issues, disaggregated by condition and age group.

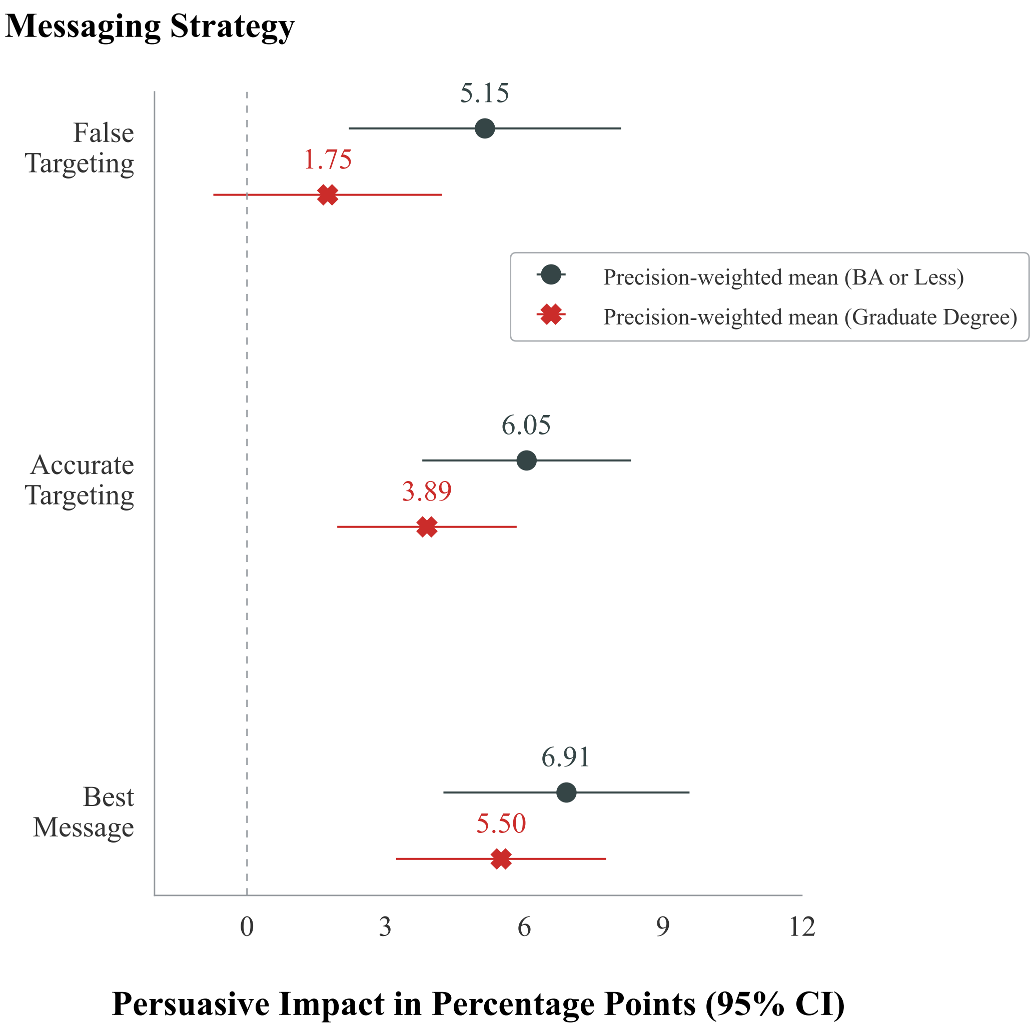

Figure S6: Precision-weighted mean of the average treatment effects across all issues, disaggregated by condition and level of education.

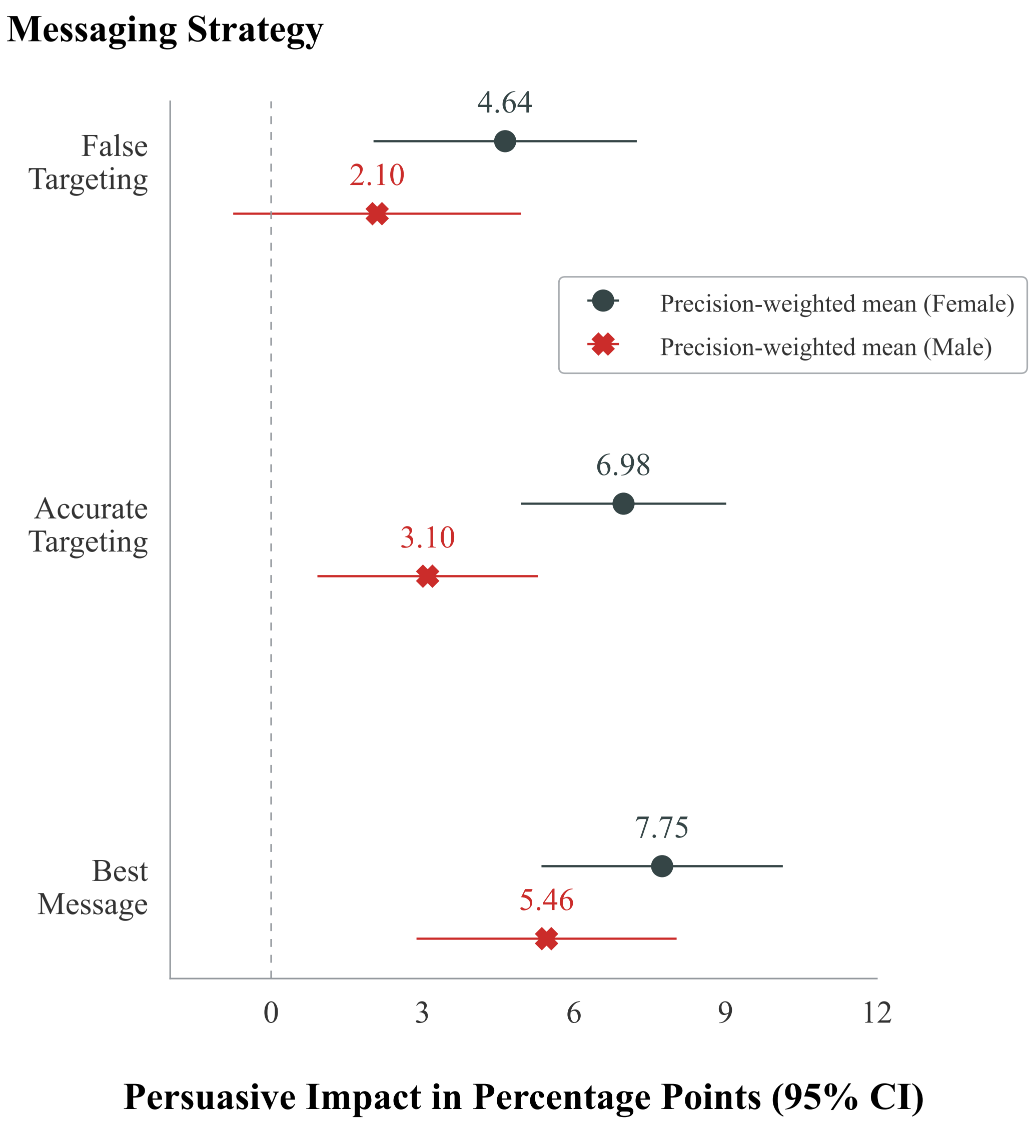

Figure S7: Precision-weighted mean of the average treatment effects across all issues, disaggregated by condition and gender group.

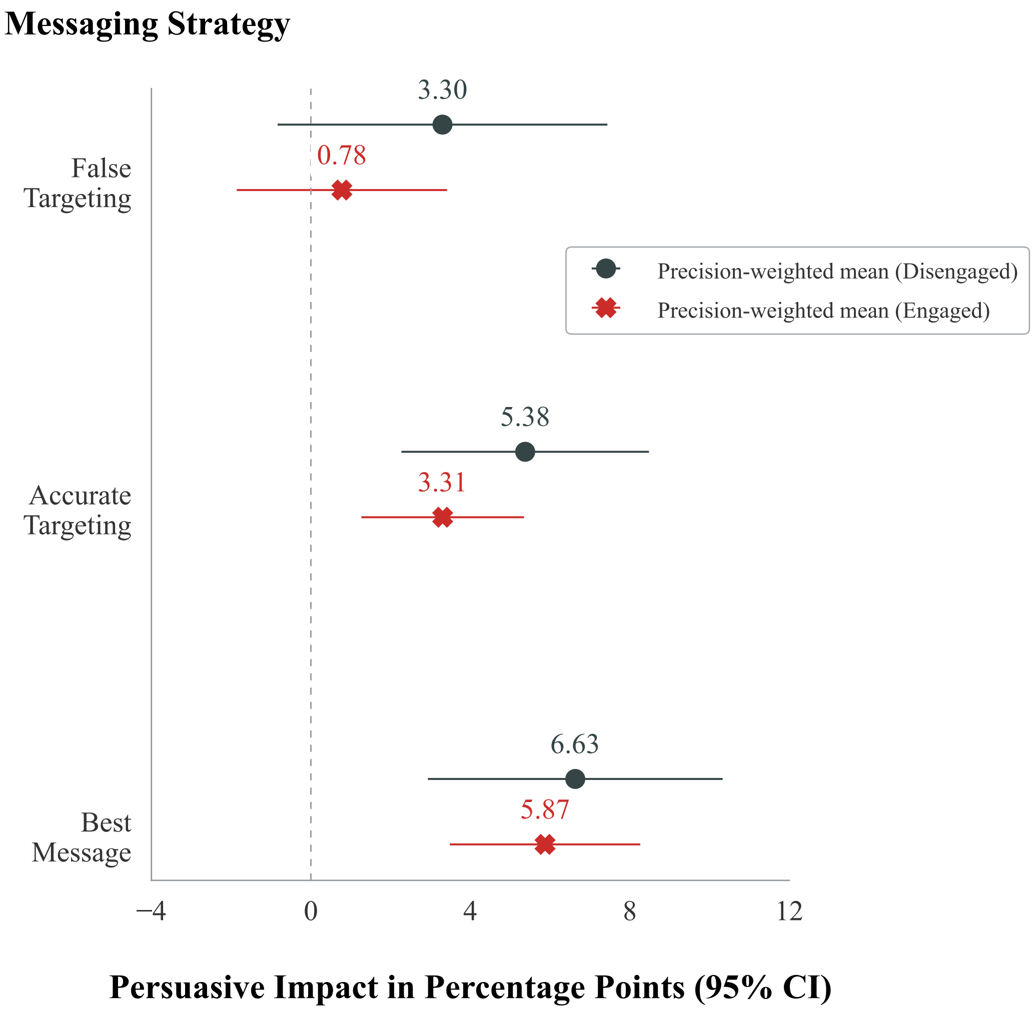

Figure S8: Precision-weighted mean of the average treatment effects across all issues, disaggregated by condition and self-reported level of political engagement.

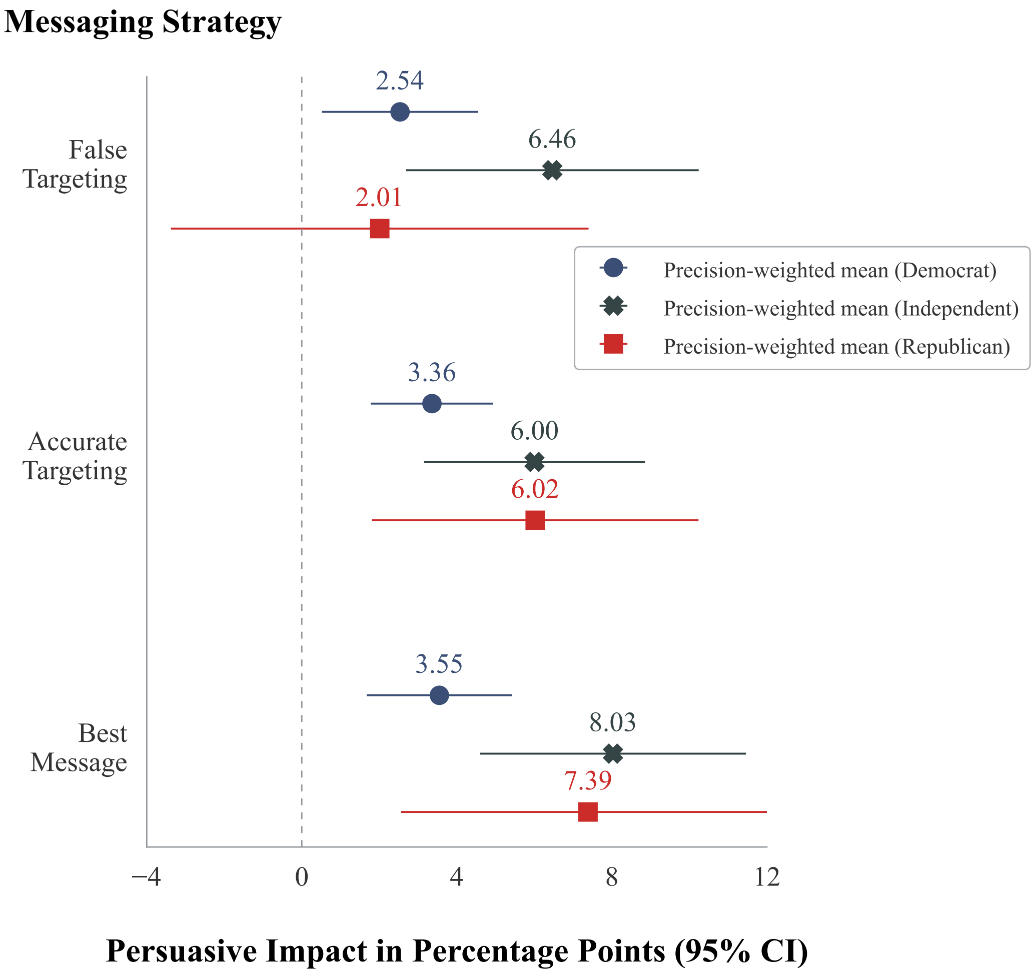

Figure S9: Precision-weighted mean of the average treatment effects across all issues, disaggregated by condition and political party.

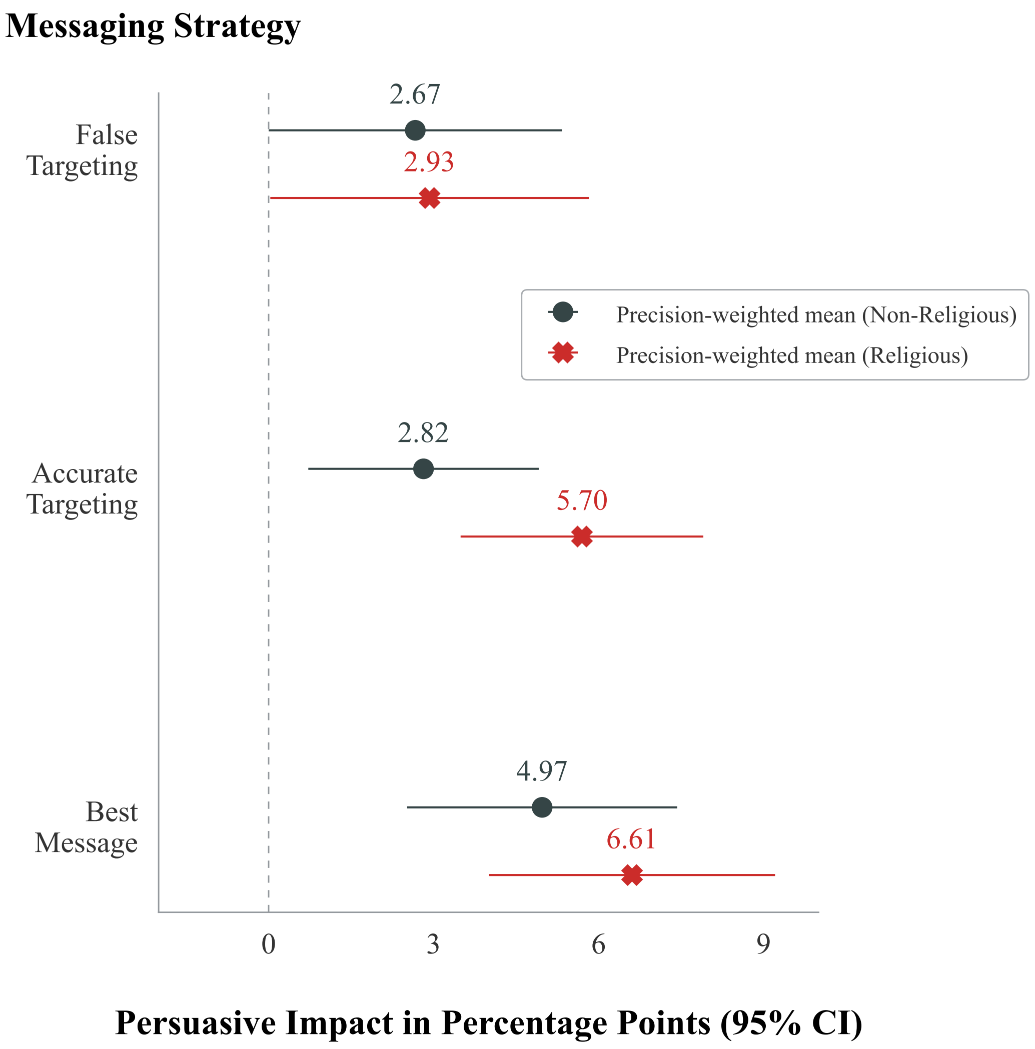

Figure S10: Precision-weighted mean of the average treatment effects across all issues, disaggregated by condition and religious beliefs.

4.3 Post-treatment Survey

After providing a dependant variable response, participants were asked to report who they thought was most likely the author of the message they were exposed to. **Table S3** shows the distribution of responses. Participants in the accurate targeting condition were 3 percentage points more likely to identify the message as AI-generated compared to the best message condition ($P = 0.012$).

Table S3: Participant perceptions of message authorship across the three treatment conditions, expressed as a proportion and in descending order.

**Author**	**Treatment Condition**		
	**False Targeting**	**Accurate Targeting**	**Best Message**
Activist	0.20	0.20	0.22
Politician	0.18	0.18	0.19
**AI**	**0.16**	**0.17**	**0.14**
Journalist	0.16	0.14	0.15
Unsure	0.09	0.09	0.09
Student	0.08	0.09	0.09
Professor	0.08	0.07	0.05
Average Person	0.05	0.05	0.05
Other	0.02	0.01	0.01

As an additional measure of construct validity, participants were also asked who they thought would find the message they were shown most compelling, in terms of similarity to themselves. Participants rated the message they were shown on a scale from "persuasive to someone very different from me" to "persuasive to someone very similar to me", where similarity was explicitly defined as sharing political and demographic attributes. This question aimed to assess if participants who received the messages tailored to their attributes actually perceived the message as likely to be compelling to someone like themselves.

The results, shown in **Figure S11**, suggest that to some extent, this was the case: participants in the accurate targeting condition who were shown messages accurately tailored to some combination of their attributes were statistically more likely to say that the messages would be most compelling to someone "somewhat similar" to themselves, compared to those who received a non-tailored "best message" (+4.3 percentage points, respectively, $P = 0.001$) or those who received a message tailored on incorrect attributes (+4.4, $P = 0.009$). Comparably, participants in the false targeting condition who were shown messages tailored to some combination of incorrect attributes were statistically more likely to say that the messages would be most compelling to someone "very different" from themselves, compared to those who received an accurately tailored message (+2.8, $P = 0.001$). Overall, however, these effects were modest, with most individuals perceiving all manner of message as broadly persuasive to individuals both similar and dissimilar to themselves.

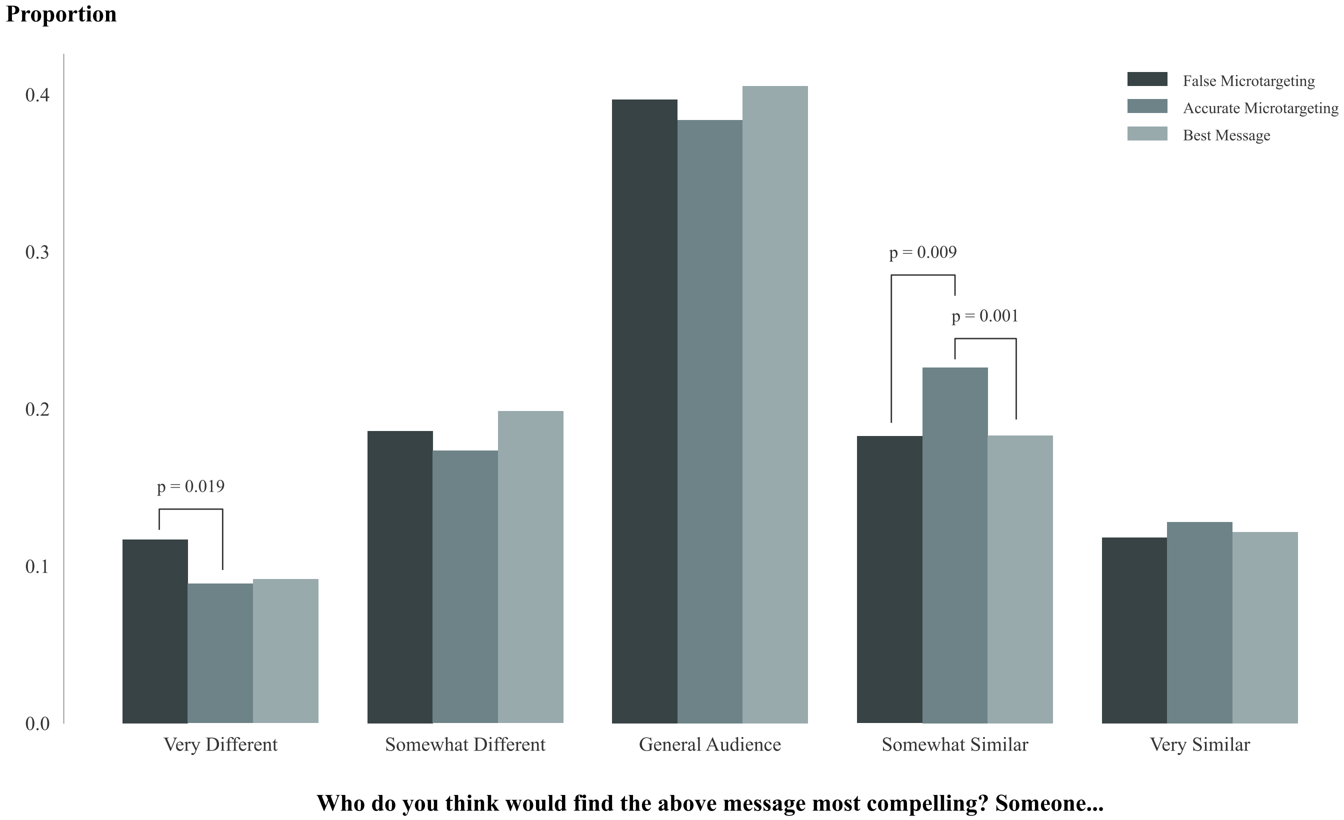

Figure S11: Participant perceptions of ideal message audience, in terms of similarity to themselves. Participants in the accurate targeting condition who were shown messages accurately tailored to some combination of their attributes were statistically more likely to say that the messages would be most compelling to someone "somewhat similar" to themselves, while participants in the false targeting condition who were shown messages tailored to some combination of incorrect attributes were statistically more likely to say that the messages would be most compelling to someone "very different" from themselves.

5 Attrition Analysis

Our web application was not set up to capture incomplete responses from participants who left before the data was sent to our database. However, Prolific retains this data. As a result, we can report that approximately 412 participants (4%) left the study before providing an outcome. Improtatntly, this is not the true *post-treatment* attrition number, since presumably a portion of those 412 left the study before being exposed to a treatment (e.g., in the first 30 seconds). However, to be conservative (and to mathematically preclude the possibility of attrition as a driving factor in our results), for our subsequent attrition simulations we use this higher 4% figure retained from Prolific.

(*Note:* To contextualize the likely *post-treatment* attrition rate, we offer data from a recent RCT experiment conducted by the lead author using:

1. the same participant recruitment platform (Prolific),
2. the same participant pre-screening criteria,
3. persuasive messages of the same length,
4. generated by the same LLM (GPT-4),
5. where ATEs were estimated using the same null control condition,
6. and where all other experimental design aspects were also the same or similar.

This experiment had a post-treatment attrition rate of **1.4%**, with no differential attrition across treatment conditions.)

5.1 Sensitivity Analysis

To investigate the possibility of differential attrition at the condition level being a driving factor in our results, we conduct a sensitivity analysis to simulate the maximum level of differential attrition we could have experienced. In particular, we take the attrition data retained from Prolific suggesting that approximately 4% of participants exited our study without providing an outcome and impute these synthetic responses into our data, such that we model the extremely unlikely case where a) every attriting participant was assigned to the microtargeting condition and b) they were all maximally persuaded by the message they saw (reporting an outcome of perfect 100).

Even had this been the case, our sensitivity analysis show that the difference between microtargeting and non-microtargeting ATE's would *still* not have been significant ((Z-statistic: 1.858, $P = 0.0643$).

5.2 Balance Checks

To offer evidence that attrition was not differential on the basis of demographic attributes, we conducted balance checks using an analysis of variance (ANOVA) approach. In particular, we conducted F-tests that fail to reject the null hypothesis that the mean covariate values are the same across all conditions.

We used binary indicators for ethnicity, gender, education level, and religious affiliation, a continuous variable for Age, and ordinal variables for political party, ideology, and engagement. We fit an OLS linear regression model for each covariate, defined as a function of the treatment condition (covariate $\sim$ condition), where the treatment condition as a categorical variable. We then performed ANOVA on each fitted model to determine if there were statistically significant differences in covariate distributions across treatment groups. The results from these analyses suggest that there was no evidence of differential attrition on the basis of these demographic traits (F-tests failed to reject the null hypothesis that the mean covariate values are the same across all conditions). The full results can be found in **Table S4**.

Table S4: F-tests for differences in mean covariate values across conditions.

Term	df	Sum Sq	Mean Sq	F Statistic	P-Value	Covariate
Condition	3.0	363.74	121.25	0.65	0.585	Age in Years
Condition	3.0	1.58	0.53	0.40	0.756	Political Party [0-4]
Condition	3.0	0.52	0.17	0.12	0.949	Political Ideology [0-4]
Condition	3.0	2.26	0.75	0.92	0.431	Political Engagement [0-3]
Condition	3.0	1.00	0.33	1.68	0.169	White [0,1]
Condition	3.0	0.11	0.04	0.15	0.930	Gender [0,1]
Condition	3.0	0.73	0.24	1.00	0.393	BA degree or less [0,1]
Condition	3.0	0.66	0.22	1.21	0.303	Religious [0,1]
